# Hydrogel and Organogel-Based Flexible Wearable Sensors: Synthesis, Sensing Mechanisms, Performance Optimization, and Applications

**DOI:** 10.3390/gels12100931

**Published:** 2026-10-15

**Authors:** Shuolei Wang, Zhiyong Xu, Mengfei Wang, Yunpeng Zhang, Wenguang Yang, Ruiqian Wang

**Affiliations:** 1 School of Electromechanical and Automotive Engineering, Yantai University, Yantai 264005, China; 19133807236@163.com (S.W.); xzy1246723667@163.com (Z.X.); 16650838208@163.com (M.W.); ytu_yangwg@163.com (W.Y.); 2 School of International Studies, Shandong Medical and Pharmaceutical University, Yantai 264005, China; zhangyunpeng0325@sdmpu.edu.cn

**Keywords:** conductive hydrogels, organohydrogels/mixed-solvent gels, composite gel materials, flexible wearable sensors, self-healing chemistry

## Abstract

Hydrogel-based flexible sensors have emerged as a promising platform at the intersection of materials science, flexible electronics, and biomedical engineering, owing to their tissue-like mechanical compliance, tunable ionic conductivity, and versatile chemical functionalizability. To translate laboratory-scale hydrogel devices into durable, reliable wearable systems, this review critically summarizes recent progress in functional hydrogels and organogels, covering literature reported up to September 2026, and organizes the discussion along the complete chain from material synthesis and characterization to wearable deployment. We first outline the synthesis principles of gel network architectures, including double networks, semi-interpenetrating networks engineered with nanosheet crosslinkers, organohydrogels employing mixed solvent systems, and mechanically interlocked topologies, followed by MXene-based composite gel materials and advanced 3D printing fabrication strategies. Six representative sensing mechanisms, namely piezoresistive, capacitive, triboelectric, thermoelectric, electrochemical, and multimodal decoupling, are then qualitatively and, where possible, quantitatively compared regarding their performance characterizations. We also discuss signal processing and readout electronics for each sensing mechanism, which are critical for wearable deployment. Dedicated sections analyze performance enhancement strategies aimed at mechanical robustness and sensitivity, and summarize self-healing chemistries along with environmental adaptation approaches, such as solvent replacement and deep eutectic solvent engineering, which are essential for long-term operational stability. We further propose a minimum reporting checklist for standardization and benchmarking and provide a systematic evaluation of long-term stability, toxicity, degradation data, leachables. Finally, we highlight application frontiers of these gel materials in health monitoring biosensing, electronic skin, and human–machine interaction, and conclude with a critical assessment of key challenges in scalable manufacturing, biocompatibility, multimodal crosstalk, energy autonomy, and environmental sustainability. A dedicated discussion of environmental impact and sustainability, covering bio-based polymers, biodegradability, recycling, and life-cycle assessment.

## 1. Introduction

### 1.1. The Rise in Flexible and Wearable Electronics

Over the past two decades, the rapid evolution of flexible and wearable electronics has fundamentally reshaped the landscape of personal healthcare, soft robotics, prosthetics, and intelligent human–machine interfaces [1,2,3,4,5,6]. Conventional rigid silicon-based devices, optimized for decades of integrated circuit miniaturization, are intrinsically mismatched with the soft, curved, and dynamically deforming surfaces of the human body. Skin tolerates strains of tens of percent during joint flexion; muscles contract by up to 20–40%; cardiac tissue cyclically deforms at approximately 10% strain more than 100,000 times per day. Any electronic platform intended for long-term intimate contact with such tissues must therefore sustain bending, stretching, twisting, and compression while maintaining stable electrical performance. This mechanical requirement has motivated an extraordinary diversification of material platforms, ranging from ultrathin flexible films and liquid-metal circuits to intrinsically stretchable polymer composites.

Among the diverse candidates explored to date, hydrogels occupy an important and rapidly growing position. A hydrogel is a three-dimensional polymer network swollen with an aqueous phase, and this simple structural definition encapsulates the properties that make hydrogels attractive for sensing: their Young’s moduli span the same range as soft biological tissues (from a few kPa for brain-mimicking matrices to several MPa for cartilage-like networks), their water-rich networks permit ion transport with conductivities tunable over orders of magnitude, and their chemical functionality can be tailored through monomer selection, crosslinking chemistry, and filler incorporation [7,8,9,10]. Crucially, unlike electronically conductive elastomers that require percolated rigid fillers, ionically conductive hydrogels conduct through mobile ions—Na^+^, Li^+^, Ca^2+^, Cl^−^—whose transport is inherently compatible with the electrochemical signaling of biological systems. This ionic-to-biological symmetry underpins the low-impedance, high-fidelity interfaces that hydrogel electrodes achieve with skin, muscle, and even internal organs.

### 1.2. From Simple Gels to Architected Functional Materials: Synthesis and Composite Design

The design space of hydrogel sensors has expanded dramatically over the past decade. Early systems relied on simple ionically conductive polyacrylamide (PAM) and polyvinyl alcohol (PVA) networks, whose mechanical weakness and environmental fragility restricted them to short-term demonstrations. Contemporary research instead embraces sophisticated architectures. Double-network (DN) hydrogels embed sacrificial bonds—ionic interactions, hydrogen bonds, or reversible covalent linkages—that fragment under load to dissipate energy while a second, stretchable network maintains structural integrity, mimicking the energy-dissipating hierarchy of natural tendon and cartilage [11]. Semi-interpenetrating networks (semi-IPN) thread linear polyelectrolytes through covalently crosslinked matrices, decoupling ionic conductivity from mechanical stiffness. At the frontier of supramolecular design, mechanically interlocked polyrotaxane gels exploit the “pulley effect” of sliding macrocycles threaded on polymer chains, achieving 2450% stretchability with stable cycling over 10,000 iterations [12].

In parallel, the functional filler library has grown from carbon nanotubes (CNTs) and graphene to two-dimensional transition metal carbides and nitrides (MXenes), liquid metals, conductive polymers such as PEDOT:PSS, and most recently magnesium boride nanostructures [13,14,15]. Each filler class offers a distinct trade-off: MXene delivers metallic in-plane conductivity with hydrophilic, hydrogen-bond-rich terminations but suffers oxidative degradation; liquid metals provide deformable, self-healing conductive domains with effectively infinite stretchability; conductive polymers add mechanical toughness and biocompatibility at the cost of lower conductivity. A central challenge in modern hydrogel design is to combine these components such that their weaknesses cancel while their strengths compound.

Yet even the most elegant architectures cannot escape three fundamental trade-offs that define the field. First, the conductivity–mechanics dilemma: stiff, densely crosslinked networks maximize strength but immobilize ions and fracture conductive pathways, whereas loose networks conduct freely but tear under modest loads. Second, the water-content paradox: the high water fraction that grants hydrogels their tissue-like softness is precisely what renders them vulnerable to freezing, evaporation, and dimensional drift. Third, the sensitivity–linearity–durability triad: signal amplification strategies such as controlled cracking boost gauge factors by orders of magnitude but sacrifice measurement range and fatigue lifetime. Every successful sensor design represents a negotiated settlement among these competing constraints. Mechanical toughness, conductivity, sensitivity, self-healing, and environmental stability are discussed in detail in Section 4 and Section 5, and the strategies reviewed there are best understood as increasingly sophisticated instruments for managing—rather than eliminating—these trade-offs.

### 1.3. Diversification of Sensing Modalities and System Intelligence

Beyond material chemistry, the sensing modality landscape has likewise diversified profoundly. Conventional piezoresistive and capacitive transduction now coexist with self-powered paradigms: triboelectric nanogenerators (TENGs) harvest ambient mechanical contact-separation cycles to generate voltage outputs without batteries, while ionic thermoelectric (iTE) devices exploit the Soret effect—selective thermodiffusion of ions under temperature gradients—to produce thermopowers exceeding 20 mV K^−1^, more than an order of magnitude above electronic Seebeck coefficients [16]. Electrochemical aptamer-based biosensors extend the detection scope from macroscopic strain to molecular analytes, continuously tracking drug pharmacokinetics in interstitial fluid. Equally transformative is the integration of machine learning: convolutional neural networks now classify gesture signals from hydrogel arrays with accuracies exceeding 92%, while deep-learning pipelines translate raw strain waveforms into gait diagnoses, sign-language sentences, and even neurological screening indicators [17].

### 1.4. Scope and Organization of This Review

**Figure 1:**
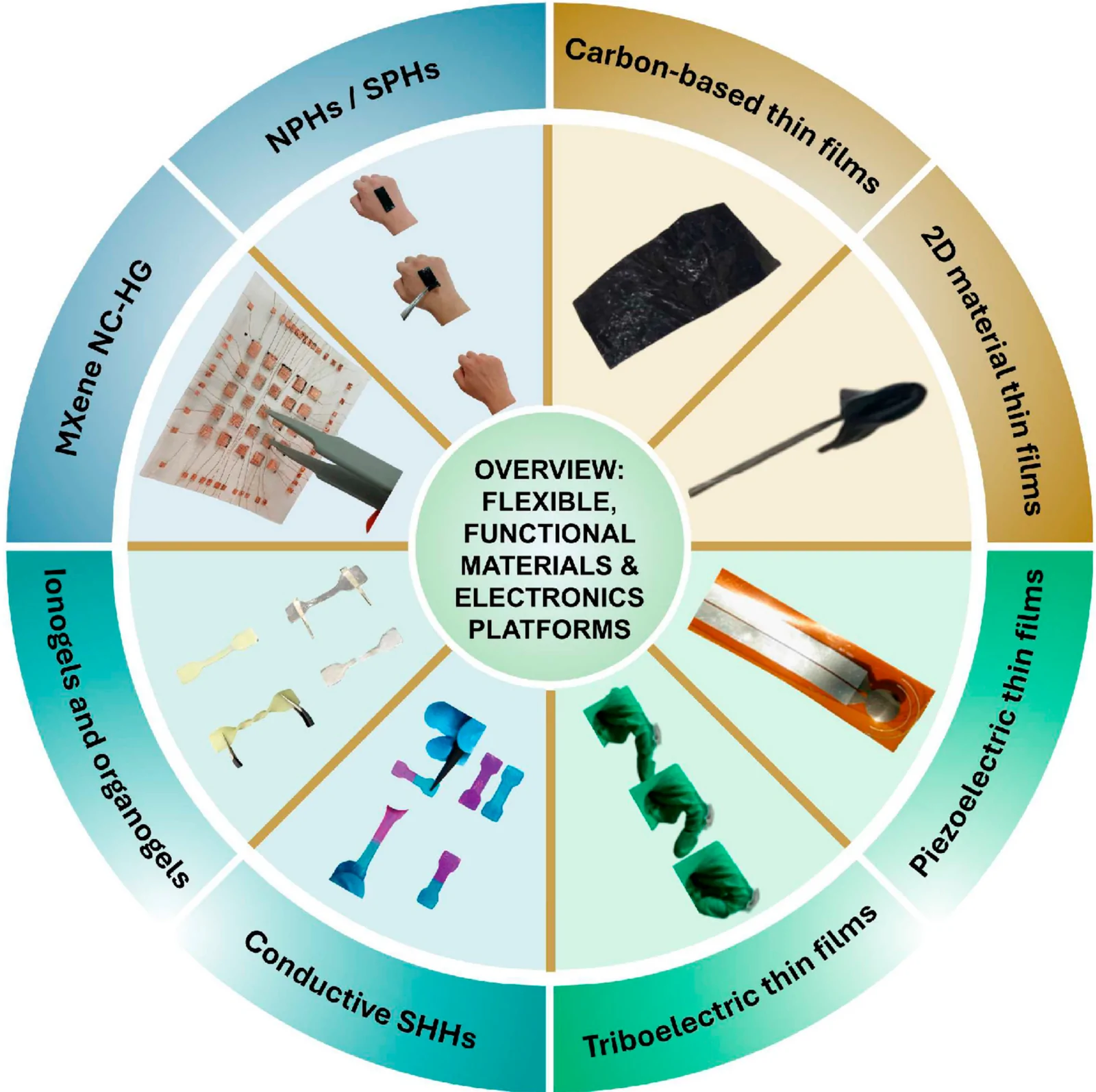
Overview of flexible functional materials and electronic device platforms. Eight representative flexible functional material systems are shown for context: carbon-based films. Reproduced with permission from Ref. [3]. Copyright *ACS Omega*. Two-dimensional material films. Reproduced with permission from Ref. [19]. Copyright *Polymers*. Piezoelectric films. Reproduced with permission from Ref. [20]. Copyright *Sensors*. Triboelectric films. Reproduced with permission from Ref. [16]. Copyright *Polymers*. Conductive self-healing hydrogels. Reproduced with permission from Ref. [18]. Copyright *Advanced Science*. Ionogels and organogels. Reproduced with permission from Ref. [21]. Copyright *Advanced Science*. MXene nanocomposite hydrogels. Reproduced with permission from Ref. [22]. Copyright *Science Advances*. Nanoporous/superporous hydrogels. Reproduced with permission from Ref. [1]. Copyright *Gels*. The core scope of this review is hydrogel- and organogel-based wearable sensors. The carbon-based, 2D, piezoelectric, and triboelectric thin-film systems are included as comparative benchmarks and complementary device components rather than as primary review subjects.

Figure 1 is presented to situate this review within the broader landscape of flexible functional materials and electronics platforms. The figure illustrates eight representative categories: carbon-based thin films, two-dimensional (2D) material thin films, piezoelectric thin films, triboelectric thin films, conductive self-healing hydrogels [18], ionogels and organogels, MXene nanocomposite hydrogels, and nanoporous/superporous hydrogels (NPHs/SPHs). However, the core scope of this review is hydrogel- and organogel-based wearable sensors. The carbon-based, 2D, piezoelectric, and triboelectric thin-film categories are included only as comparative benchmarks and complementary device components; they are not reviewed comprehensively.

The survey is organized along the full chain from material design to wearable deployment. The structure maps primarily to the hydrogel and organogel core systems, while the other material platforms appear only where they provide useful comparison or complementary function. Section 2 discusses material design principles and fabrication strategies of hydrogel systems, covering network architectures that correspond to conductive self-healing hydrogels, ionogels, and organogels, as well as MXene-reinforced nanocomposite hydrogels and advanced 3D printing fabrication. Section 3 dissects six representative sensing mechanisms. In this section, porous microstructures, piezoelectric thin films, triboelectric thin films, carbon-based conductive films, and 2D conductive films are mentioned as structural or functional components rather than as the main subject of the review. Section 4 analyzes performance enhancement strategies targeting both mechanical robustness and sensing sensitivity, where porous engineering and 2D material reinforcement are discussed as approaches applied to hydrogel systems. Section 5 reviews self-healing chemistries and environmental adaptation approaches that determine long-term operational stability of hydrogel and organogel sensors. Section 6 surveys application frontiers in health monitoring biosensing, electronic skin, and human–machine interaction. Section 7 concludes with critical challenges and future perspectives.

### 1.5. Literature Search and Scope

This review is a critical narrative review rather than a PRISMA-guided systematic review. The literature search was performed in September 2026. Literature was retrieved from Web of Science, Scopus, PubMed, and Google Scholar using combinations of keywords including “hydrogel”, “organogel”, “ionogel”, “flexible wearable sensor”, “self-healing”, “MXene”, “triboelectric”, “thermoelectric”, “electrochemical”, and “3D printing”. The search covered peer-reviewed publications up to September 2026. Priority was given to original research and review articles; non-peer-reviewed sources were used only for background. The inclusion criteria were as follows: studies had to report original experimental data or comprehensive reviews on hydrogel or organogel materials for flexible wearable sensors; studies had to be published in peer-reviewed journals; and studies had to be written in English. The exclusion criteria were as follows: conference abstracts, non-peer-reviewed preprints, and studies that did not report sufficient experimental details. This review is intended as a critical narrative synthesis rather than a systematic review, and the literature coverage period reflects the rapid growth of the field from 2025 to 2026.

### 1.6. Distinctiveness and Novelty of This Review

Several reviews have addressed hydrogels, organogels, and wearable sensors. However, most existing reviews focus on a single material class, a single sensing mechanism, or a single application domain. This review provides an integrated framework that connects material synthesis, network architecture, sensing mechanisms, performance optimization, self-healing chemistry, environmental adaptation, and wearable deployment in one coherent discussion. The specific contributions of this review are fourfold.

First, it organizes hydrogel and organogel design along the complete chain from molecular network engineering to device-level wearable integration, rather than treating synthesis, sensing, and application as separate topics. Second, it compares six representative sensing mechanisms, namely piezoresistive, capacitive, triboelectric, thermoelectric, electrochemical, and multimodal decoupling, and discusses their trade-offs in sensitivity, range, response speed, power, and stability. Third, it emphasizes the coupling between mechanical robustness and sensing sensitivity, and it treats self-healing and environmental stability as two faces of the same long-term operational problem. Fourth, it highlights emerging application frontiers in health monitoring, electronic skin, human–machine interaction, soft robotics, and AI-assisted signal interpretation.

This integrated perspective distinguishes the present review from previous publications and provides a framework for future material and device co-design. In particular, the review does not merely catalogue recent progress; it identifies the fundamental trade-offs that constrain hydrogel sensors, including the conductivity-mechanics dilemma, the water-content paradox, and the sensitivity-linearity-durability triad. It also discusses how these trade-offs are negotiated through network architecture, composite design, solvent engineering, self-healing chemistry, and signal processing. Therefore, this review offers both a comprehensive overview and a design-oriented framework for researchers working on hydrogel and organogel wearable sensors.

## 2. Synthesis and Fabrication of Functional Gels

### 2.1. Design and Synthesis of Physical and Chemical Gel Networks

The mechanical and electrical performance of hydrogel sensors is governed fundamentally by network architecture, which determines how stress redistributes, how cracks arrest, and how ions migrate under deformation. Four architectural families dominate the current literature: double networks, interpenetrating networks, nanocomposite heterostructures, and solvent-engineered organohydrogels.

**Figure 2:**
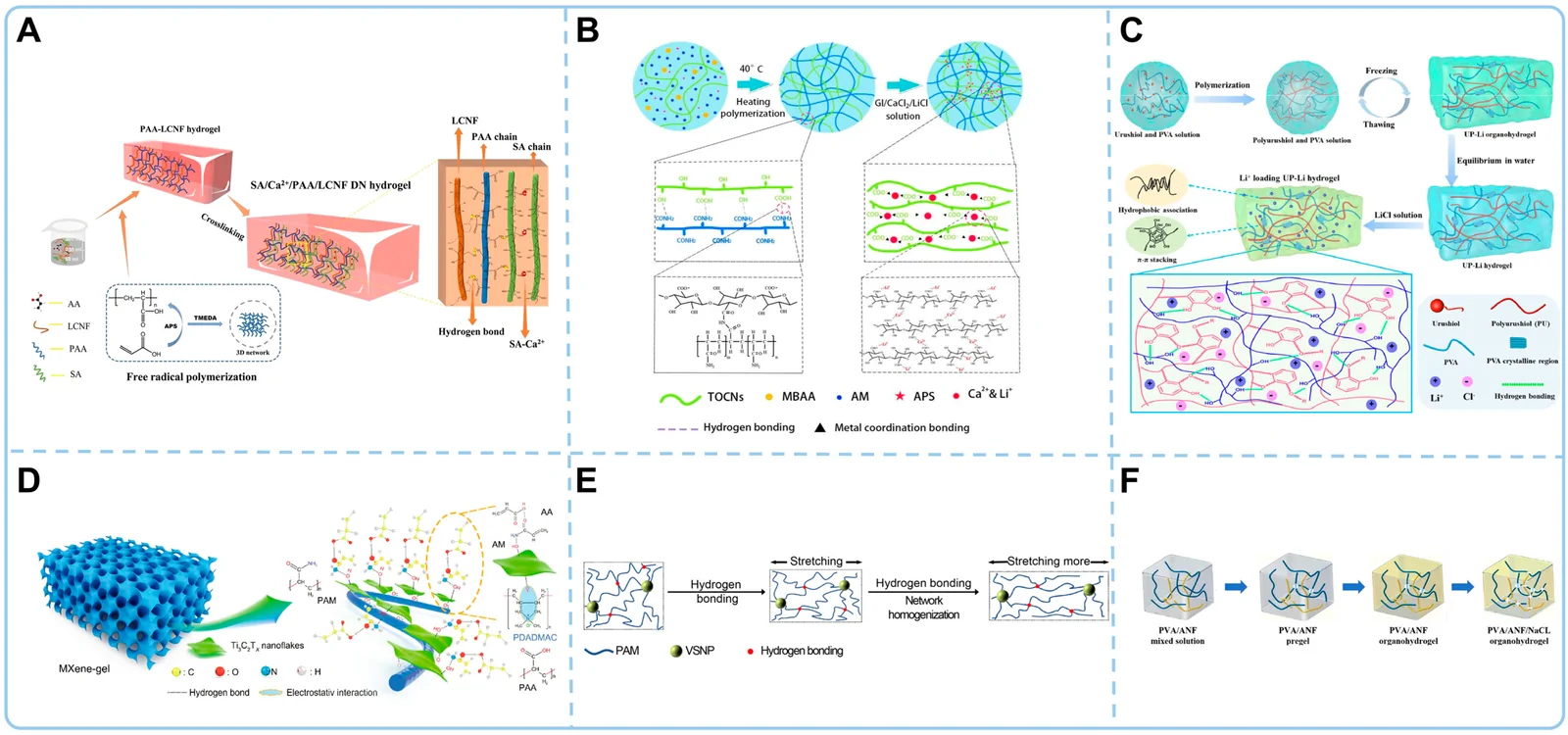
Network structure evolution of hydrogel sensors: (**A**) Double-network structure. Reproduced with permission from Ref. [23]. Copyright *Polymers*. (**B**) Ionic crosslinked network. Reproduced with permission from Ref. [24]. Copyright *Gels*. (**C**) Ion-conductive double network. Reproduced with permission from Ref. [25]. Copyright *Polymers*. (**D**) Semi-interpenetrating network. Reproduced with permission from Ref. [26]. Copyright *Advanced Science*. (**E**) MXene-hydrogel heterostructure. Reproduced with permission from Ref. [22]. Copyright *Science Advances*. (**F**) Organohydrogel. Reproduced with permission from Ref. [27]. Copyright *Gels*.

Double-network design. The archetypal DN strategy pairs a stiff, brittle first network with a soft, stretchable second network. Under load, the first network fragments into microcracks that dissipate energy, while the second network bridges the fragments and maintains macroscopic integrity. Representative implementations illustrate the versatility of this concept. Polyacrylic acid (PAA)/LCNF/SA hydrogels reinforce an alginate-polyacrylic acid dual network with lignocellulose nanofibrils, exploiting the rigid biopolymer as both a mechanical scaffold and hydrogen-bond mediator to achieve enhanced toughness for strain sensing (Figure 2A) [23]. Ionically crosslinked dual networks comprising polyelectrolyte complexes deliver high mechanical strength, electrical conductivity, water retention, and frost resistance simultaneously—a quartet of properties that single-network gels rarely achieve—satisfying the multifunctional demands of wearable strain sensors across seasons (Figure 2B) [24]. Urushiol-based ionic conductive DN hydrogels demonstrate that even natural phenolic building blocks extracted from lacquer trees can be reconstituted into ultrastrong and tough matrices suitable for flexible strain sensing, an instructive example of biomass valorization in functional materials (Figure 2C) [25]. Quantitatively, well-designed DN systems reach fracture energies of 1000–1500 J m^−2^—approaching articular cartilage—whereas single networks typically fail below 10 J m^−2^.

Interpenetrating network topologies. Interpenetrating network (IPN) and semi-interpenetrating network (semi-IPN) architectures offer an alternative route to decouple conductivity from mechanics. In a semi-IPN, a preformed linear polymer threads through a covalently crosslinked network without being chemically bound to it, so the linear chains can relax under strain while the crosslinked skeleton bears load. When MXene nanosheets are introduced as multifunctional junctions into a semi-IPN of PDADMAC and PAM, the resulting water-retentive gels sustain ultra-stable strain sensing with real-time gesture recognition capability, because the 2D fillers both bridge chains mechanically and provide percolated electronic pathways (Figure 2D) [26]. Heterostructured configurations push this concept to its logical extreme: mixed-dimensional MXene–hydrogel heterostructures couple vinyl-hybrid-silica-nanoparticle (VSNP)-modified PAM hydrogels with 2D MXene through polypyrrole nanowire nano-bridging layers, achieving an extraordinary working range of 2800%, ultrafast response within 90 ms, and resilience within 240 ms, alongside tunable sensing mechanisms and controlled crack generation (Figure 2E) [22]. The nano-bridging strategy is significant because it addresses the perennial interface problem of 2D/3D composites: without compliant interlayers, stress concentrates at the hydrogel–nanosheet boundary and initiates premature failure.

Organohydrogels and solvent engineering. For operation in demanding environments, solvent engineering replaces part of the network water with an organic phase while preserving ion-transport pathways. A representative PVA/ANF/NaCl composite organohydrogel is fabricated through a three-step protocol—freeze–thaw crosslinking, solvent exchange, and NaCl impregnation—in which each component performs a discrete role: 2 wt% aramid nanofibers significantly enhance the mechanical performance by hydrogen-bonded reinforcement of the PVA network, 0.5 M NaCl achieves the optimal mechanical–electrical balance by supplying mobile ions without over-plasticizing the matrix, and the solvent-exchanged phase suppresses freezing and dehydration (Figure 2F) [27]. The resulting sensor monitors distance, pressure, and strain with high cyclic stability, and a coupled triboelectric nanogenerator extends the platform into a fully self-powered handwriting recognition system achieving 95% accuracy on a test set—demonstrating that organohydrogel architectures can support not only passive sensing but also energy harvesting and machine-learning-assisted signal interpretation. Complementary ANF-reinforced PVA organohydrogels employing DMSO/H_2_O binary solvents preserve ionic conductivities of 1.1–34.3 S m^−1^ across a −50 to 60 °C window, confirming the generality of solvent engineering for wide-temperature operation [21]. Beyond these canonical designs, tough DN hydrogels with rapid self-reinforcement and low hysteresis based on highly entangled networks have demonstrated fatigue thresholds exceeding 1 kJ m^−2^ [28], while toughening strategies via polyelectrolytes, supramolecular assemblies, and granular architectures continue to enrich the design toolbox [29,30,31,32]. Collectively, these architectures illustrate a coherent trend: from single networks to hierarchical, multiscale, and mechanically interlocked topologies, each generation transferring additional energy-dissipation mechanisms into the gel matrix.

Two design principles emerge from this architectural survey. The first is hierarchical dissipation: architectures that distribute energy release across multiple length scales—sacrificial bonds at the molecular level, phase-separated domains at the mesoscale, and entangled or interlocked chains at the network level—consistently outperform single-mechanism designs across all four performance axes of strength, toughness, fatigue resistance, and recoverability. The second is functional multiplexing: the most cited recent systems no longer treat the network merely as a mechanical substrate but load it with additional roles—water retention, ionic transport, self-healing, and stimulus responsiveness—through deliberate choice of crosslinking chemistry. Network architecture, in other words, has evolved from a purely mechanical concern into the master variable that couples every downstream function of the sensor.

The network architecture discussed above also determines long-term stability. Water loss, swelling, drying, freezing, chemical degradation, and changes in conductivity are not independent of material design. For example, covalent crosslinks resist swelling but can embrittle the network, whereas reversible physical crosslinks tolerate deformation but may rearrange under sustained load. Solvent-engineered organohydrogels suppress freezing and drying, but their conductivity and mechanical properties depend strongly on solvent composition. Salt-loaded networks maintain ionic conductivity at low temperature but can deliquesce and drift dimensionally in humid environments. Deep eutectic solvent systems eliminate evaporative loss but may weaken hydrogen-bond networks and require additional dynamic crosslinking. These connections are revisited in Section 5 and Section 7.1.

### 2.2. Synthesis and Characterization of Composite and Hybrid Gel Materials

Since the isolation of Ti_3_C_2_T_x_ MXene in 2011, this 2D material has become a cornerstone filler for functional hydrogels owing to its metallic in-plane conductivity (up to ~10^4^ S cm^−1^ in solution-cast films), hydrophilic surface terminations (–OH, –O, –F) that hydrogen-bond with polymer chains, and solution processability compatible with aqueous gelation chemistry.

**Figure 3:**
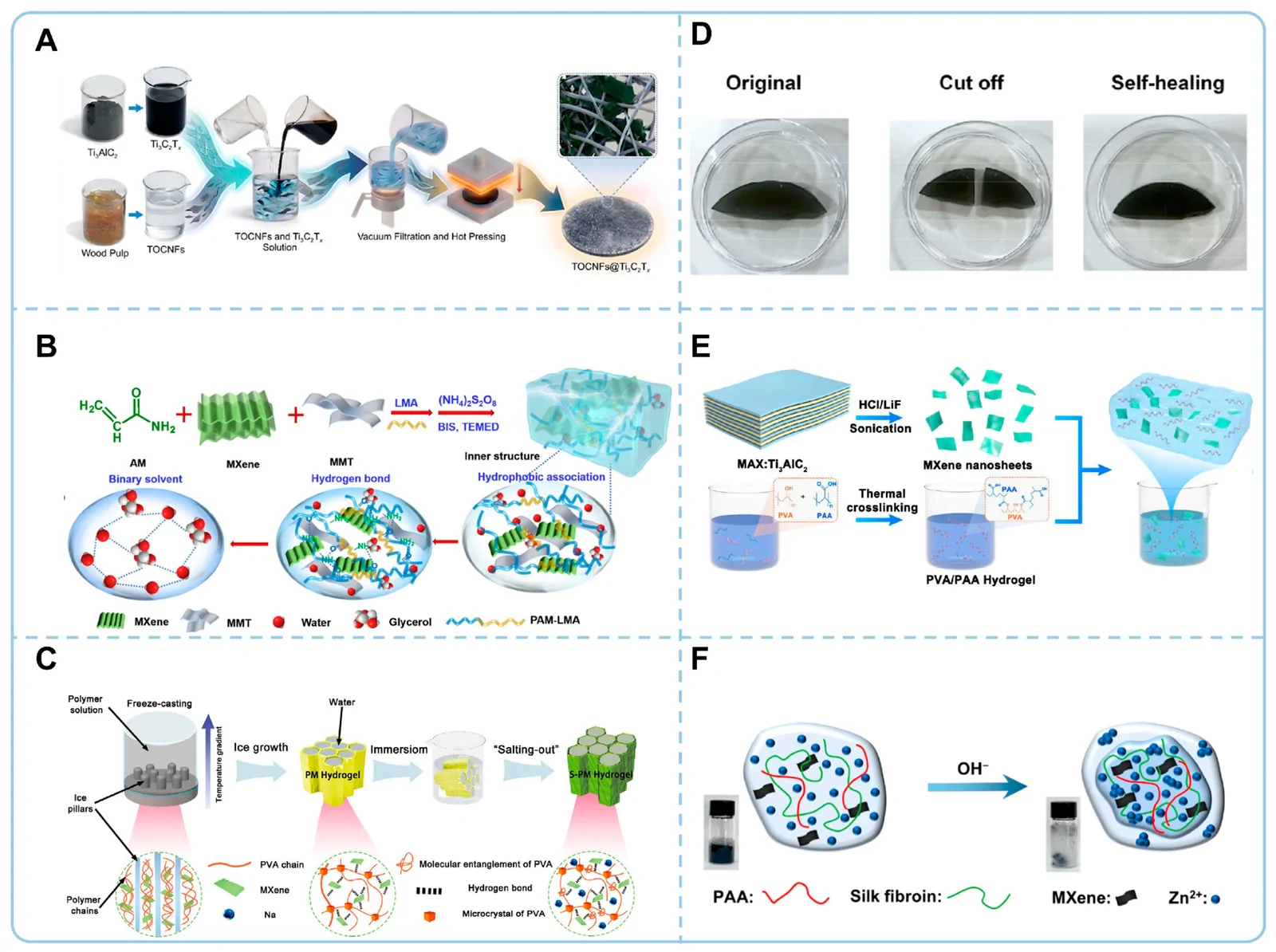
Functionalization strategies of MXene composite hydrogels: (**A**) MXene nanosheet-based nacre-mimetic architecture. Reproduced with permission from Ref. [33]. Copyright *Sensors*. (**B**) Conductivity enhancement. Reproduced with permission from Ref. [37]. Copyright *Gels*. (**C**) Mechanical reinforcement. Reproduced with permission from Ref. [38]. Copyright *Advanced Science*. (**D**) Self-healing performance. Reproduced with permission from Ref. [44]. Copyright *Polymers*. (**E**) 3D printing applications. Reproduced with permission from Ref. [45]. Copyright *Sensors*. (**F**) Stimulus-responsive sensing. Reproduced with permission from Ref. [46]. Copyright *Gels*.

Foundational architectures and etching chemistry. The nacre-inspired TOCNFs@Ti_3_C_2_T_x_ composite films establish the “brick-and-mortar” architecture—hard MXene platelets glued by soft cellulose nanofibers—that underpins subsequent hydrogel designs (Figure 3A) [33]. Etching chemistry critically determines downstream performance: HF etching yields small, defective flakes with high oxidation propensity; the minimally intensive layer delamination (MILD) route using HCl/LiF produces larger, less-defective flakes; and electrochemical etching offers the most benign surfaces but at reduced yield [34,35,36]. Flake size governs percolation threshold, defect density controls long-term conductivity retention, and termination chemistry dictates interfacial adhesion—three parameters that must be co-optimized for stable composite gels.

Conductive and mechanical enhancement. For conductive enhancement, MXene/MMT composite hydrogels exploit synergistic assembly of MXene nanosheets and montmorillonite clay platelets within a PAM-LMA matrix, where binary water/glycerol solvents and hydrogen-bond-rich interfaces yield highly sensitive wearable sensors and flexible supercapacitors (Figure 3B) [37]. Mechanical reinforcement strategies include directional freeze-casting combined with salting-out: leveraging the Hofmeister effect, layered PVA/MXene hydrogels achieve tensile strengths up to 2.87 MPa with antiswelling behavior, retaining structure and strength after 7 days of water immersion with only 6% resistance change (Figure 3C) [38]. Interface engineering through hydrogen bonding, coordination chemistry, and polymer grafting further optimizes stress transfer between MXene and polymer chains [39,40], while oxidation-resistant designs incorporating ascorbic acid scavengers or PVA passivation layers extend the operational lifetime of MXene-based wearables from weeks toward months [41,42,43].

Functional diversification. Functional diversification of MXene gels proceeds along three axes. Self-healing MXene/PVA/PAM hydrogels with dynamic borate ester bonds recover both mechanical and electrical properties after cutting, enabling reliable long-term health monitoring (Figure 3D) [44]. Three-dimensional printing of Ti_3_C_2_T_x_–hydrogel copolymer inks yields high-precision sensing architectures that track biomechanical processes at anatomical locations impossible to address with cast gels (Figure 3E) [45]. Stimuli-responsive systems, exemplified by injectable PAA-Zn-silk fibroin-MXene hydrogels, exploit pH-triggered network compaction and Zn^2+^ coordination for multifunctional capacitive strain sensing, with injectability enabling minimally invasive deployment (Figure 3F) [46]. Recent developments extend this palette considerably: muscle-inspired anisotropic MXene gels sustain low-temperature tolerant sensor arrays by aligning MXene along thermoreversible agar/PAM networks [47]; bioinspired cellulose-integrated MXene hydrogels combine multifunctional sensing with electromagnetic interference shielding [48]; and non-swelling MXene nanocomposite gels preserve percolation architecture during prolonged immersion for underwater strain sensing [49,50].

Two unresolved concerns temper this progress. Oxidative degradation remains the Achilles heel of MXene composites: surface Ti atoms oxidize progressively in oxygenated water, converting metallic terminations to insulating TiO_2_ and degrading conductivity over days to weeks. Passivation strategies—ascorbate scavenging, PVA hydrogen-bond blankets, lyophilization for storage—extend lifetimes but add processing complexity, and a universally accepted stabilization protocol has yet to emerge. Separately, the biological fate of MXene flakes remains incompletely characterized: cytotoxicity depends strongly on dose, flake size, and surface chemistry, and chronic-exposure data for skin-contact applications lag behind device innovation. Addressing stability and safety in tandem, rather than treating them as post hoc checkboxes, will largely determine whether MXene hydrogels transition from high-impact demonstrations to certified wearable products. The trajectory of the field thus mirrors its maturation: from conductivity demonstrations toward multifunctional, environment-robust, and application-targeted systems that satisfy these emerging constraints. Comparison with other emerging conductive materials. MXene is discussed in greater detail in this review because it uniquely combines metallic in-plane conductivity, hydrophilic surface terminations, and aqueous processability, which makes it particularly compatible with hydrogel chemistry. However, MXene is not the only emerging conductive material for hydrogel sensors. Carbon nanotubes and graphene offer mature chemistry, high conductivity, and low cost, but their rigid percolation networks can reduce stretchability and their dispersion in aqueous media remains challenging. Liquid metals provide deformable, self-healing conductive domains with effectively infinite stretchability, but their long-term biodistribution and encapsulation requirements remain unresolved. Conductive polymers such as PEDOT:PSS offer biocompatibility and mechanical toughness, but their conductivity is lower than that of MXene and their stability under repeated deformation requires improvement. Carbon dots and fluorescent quantum dots have recently emerged as multifunctional fillers that combine optical sensing, drug delivery, pH sensing, nanozymatic activity, and UV shielding with hydrogel matrices. These materials broaden the functional palette of hydrogel sensors beyond electrical transduction and are therefore highly relevant to future multimodal wearable systems. Therefore, MXene should be regarded as a representative but not exclusive emerging conductive material for hydrogel sensors. Carbon dots and fluorescent quantum dots have recently emerged as multifunctional fillers that combine optical sensing, drug delivery, pH sensing, nanozymatic activity, and UV shielding with hydrogel matrices. Ganguly and Margel reviewed fluorescent quantum dot-based hydrogels for multimodal biosensing [51], and Marvi et al. demonstrated multifunctional carbon dots confined in situ within hydrogels for optical communication, drug delivery, pH sensing, nanozymatic activity, and UV shielding [52]. These materials broaden the functional palette of hydrogel sensors beyond electrical transduction and are therefore highly relevant to future multimodal wearable systems.

### 2.3. Advanced 3D Printing and Additive Manufacturing of Gels

**Figure 4:**
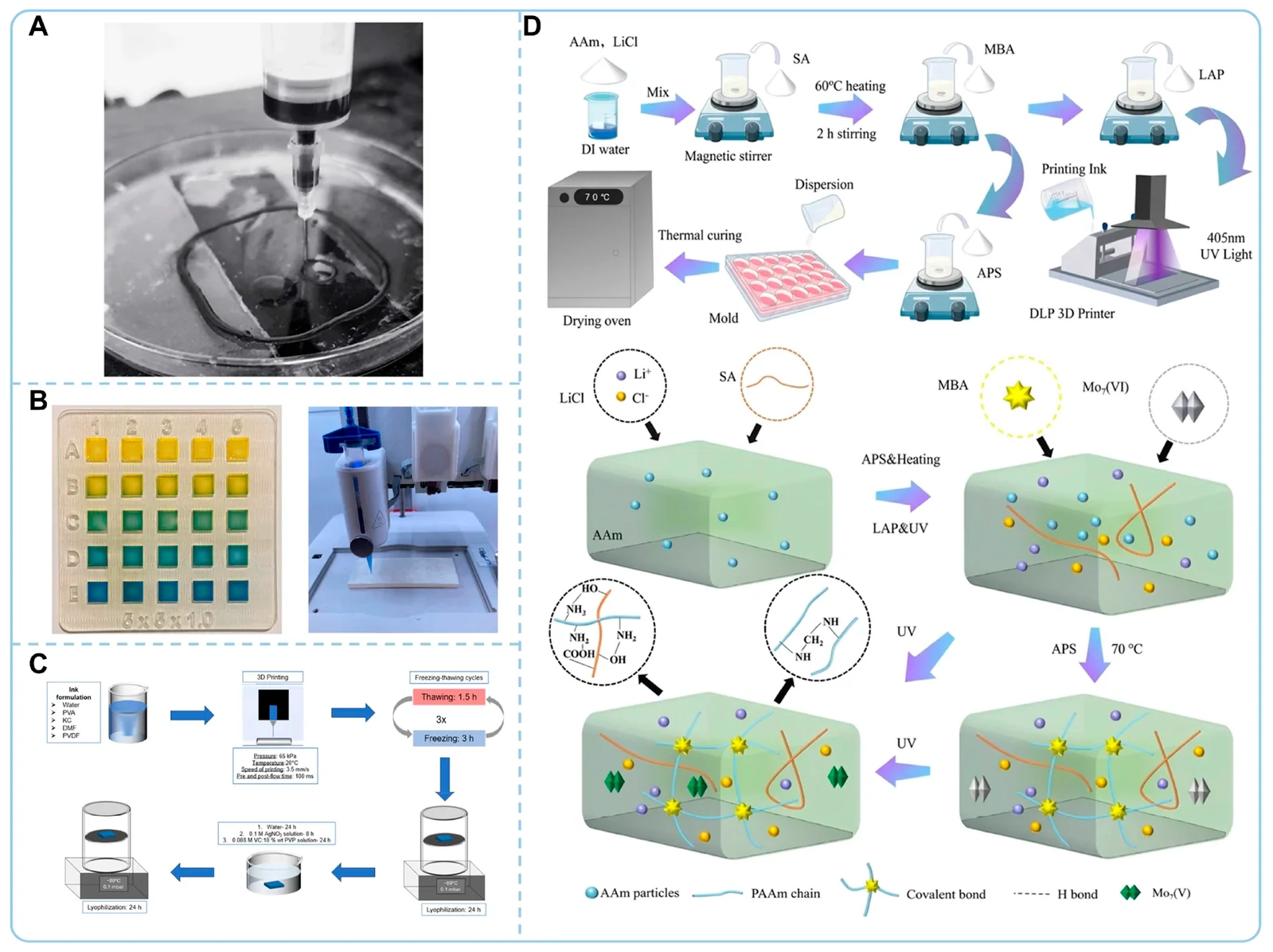
3D printing fabrication of hydrogel sensors: (**A**) Extrusion-based direct ink writing printing. Reproduced with permission from Ref. [53]. Copyright *Gels*. (**B**) DLP printed salivary pH sensor. Reproduced with permission from Ref. [56]. Copyright *Sensors*. (**C**) Conductivization via freeze–thaw post-treatment. Reproduced with permission from Ref. [61]. Copyright *Gels*. (**D**) Printing of dual-curable electronic skin. Reproduced with permission from Ref. [57]. Copyright *Gels*.

Additive manufacturing has revolutionized hydrogel sensor fabrication by enabling customized geometries, multi-material integration, spatially programmed functionality, and rapid prototyping—capabilities that casting cannot provide (Figure 4).

Extrusion-based direct ink writing. Direct ink writing (DIW) extrudes shear-thinning inks through nozzles of 100–400 μm, building structures layer-by-layer. The printability–biocompatibility trade-off is exemplified by alginate–carbon composite hydrogels, where systematic cytotoxicity evaluations confirm that the carbon filler loading required for conductivity remains within biocompatible limits, establishing a design window for skin-contact sensors (Figure 4A) [53]. Print fidelity is governed by ink rheology: the storage modulus must exceed a critical value to support overhanging features, shear-thinning behavior ensures smooth extrusion, and rapid modulus recovery after deposition prevents filament slumping [54,55]. Formulation strategies including nanoclay thickeners, gelatin yield-stress additives, and nanoparticle jamming have brought DIW hydrogel resolution into the tens-of-micrometers regime.

Digital light processing. Digital light processing (DLP) photopolymerization offers superior resolution (down to ~10 μm) and smooth surfaces by projecting patterned UV light into photocurable resin baths. A DLP-printed hydrogel sensor for rapid colorimetric salivary pH detection illustrates how formulation chemistry translates directly into sensing function: pH-indicator moieties copolymerized into the ionic network report local acidity through visible color shifts within seconds (Figure 4B) [56]. Multi-component PAAm/SA/ammonium molybdate/LiCl inks combine photo-thermal dual curing with ionic conductivity, yielding e-skin and information-encryption devices through spatially programmed gelation—405 nm UV exposure defines geometry while subsequent thermal initiation completes crosslinking of the Mo(VI)-containing network, and LiCl provides the ionic conduction (Figure 4D) [57]. Resolution considerations for photocurable systems include photoinitiator selection (LAP with 405 nm absorption vs. Irgacure 2959 with 365 nm), oxygen inhibition management through inert blanketing or amine additives, and light-penetration-depth calibration that sets the minimum feature size [58,59,60].

Post-processing functionalization. Post-processing routes substantially enrich functionality beyond what direct printing achieves. Freeze–thaw cycling of printed PVA/KC constructs followed by AgNO_3_ immersion, vitamin-C reduction, and lyophilization generates hierarchically porous conductive scaffolds with silver-decorated pore walls—effectively combining 3D geometry with metallic conductivity through solution metallization (Figure 4C) [61]. Complementary strategies include sacrificial templating with sugar or ice crystals for gradient porosity, embedded 3D printing of ionogel circuits within elastomer sheaths, and multi-material printhead switching for integrated sensing arrays [62,63]. The convergence of high-resolution printing and post-functionalization points toward a manufacturing paradigm in which sensor geometry, composition, and microstructure are independently programmable—a capability that conventional casting fundamentally cannot deliver.

Achieving these capabilities at manufacturing grade, however, demands attention to defects that laboratory demonstrations often overlook. Printed structures inherit anisotropy from layer-wise deposition: interlayer bonding is typically weaker than in-plane cohesion, and delamination under cyclic strain initiates preferentially at layer interfaces. Ink-gelation kinetics impose a second constraint—premature gelation clogs nozzles while delayed gelation causes filament sag—necessitating residence-time windows that vary with each formulation. Finally, batch-to-batch consistency of rheological and electrical properties, routinely reported as within a few percent in academic studies, remains substantially harder to maintain over thousands of prints. Inline optical inspection, closed-loop pressure control, and standardized ink characterization protocols are accordingly receiving growing attention as the community moves from prototyping toward reproducible fabrication.

## 3. Characterization of Sensing Mechanisms and Computational Understanding

**Figure 5:**
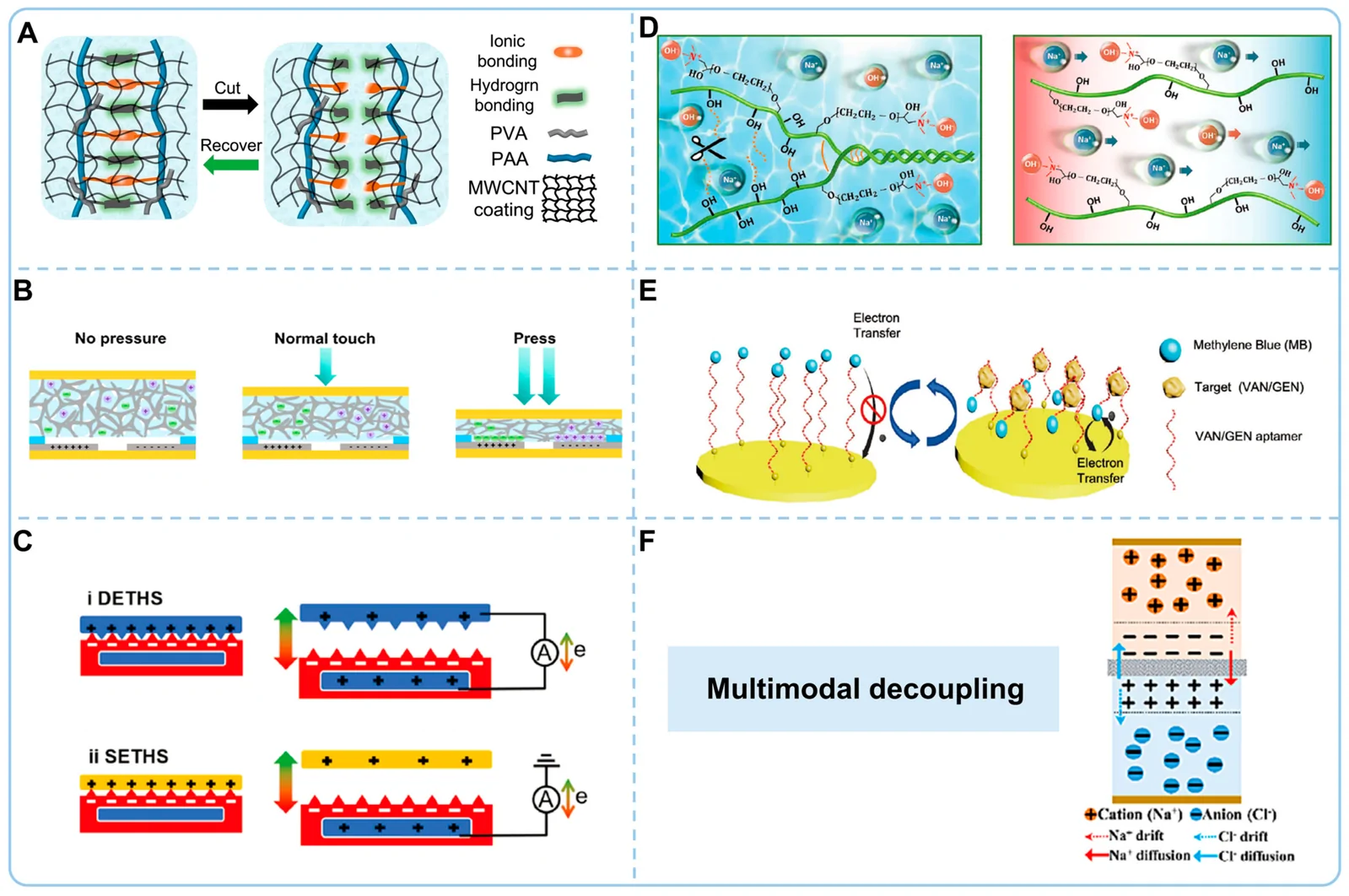
Six sensing mechanisms of hydrogel-based sensors: (**A**) Piezoresistive sensing based on surface conductive network micropatterns and controllable microcrack propagation. Reproduced with permission from Ref. [64]. Copyright *Gels*. (**B**) Capacitive sensing based on compressible microstructured three-dimensional porous ionogel dielectric layer. Reproduced with permission from Ref. [65]. Copyright *Micromachines*. (**C**) Triboelectric sensing based on micro-pyramid patterned ionic hydrogel operating in dual-electrode and single-electrode TENG modes. Reproduced with permission from Ref. [66]. Copyright *Advanced Science*. (**D**) Thermometric sensing array based on ionic thermoelectric effect with high thermopower from Soret effect. Reproduced with permission from Ref. [67]. Copyright *Advanced Science*. (**E**) Electrochemical aptamer biosensing integrated with hydrogel microneedle array. Reproduced with permission from Ref. [68]. Copyright Advanced Science. (**F**) Multimodal signal decoupling via tailored ion transport engineering of stacked hydrogels. Reproduced with permission from Ref. [69]. Copyright *Advanced Science*. A quantitative comparison of these mechanisms is provided in Table 1. A comprehensive comparison that includes working range, stretchability, durability, and typical applications is provided in Table 4. A discussion of standardization and benchmarking protocols is provided in Section 3.8, and the proposed checklist is summarized in Table 3. Signal processing and readout electronics are discussed in Section 3.9.

The transduction mechanism is the functional heart of any hydrogel sensor: it determines the sensitivity ceiling, the dynamic range, the power budget, the response speed, and—critically—the failure modes of the device. Unlike rigid sensors whose mechanisms are well established, hydrogel transduction exploits the unique physics of ion-containing soft networks, where mechanical deformation, molecular recognition, temperature gradients, and interfacial charge transfer all modulate measurable electrical signals. A hydrogel sensor is, moreover, a multi-physics object by construction: its ionic conductivity responds simultaneously to geometry, ion concentration, ion mobility, and interfacial impedance, and every successful mechanism design is at bottom a strategy for making one of these variables dominate the others. Six mechanisms dominate the current literature, and they are best understood not as isolated options but as complementary tools whose selection depends on the target application, as summarized in Figure 5 and Table 1. The distinction between ionic, electronic, and mixed conduction is important because the charge-transport mechanism strongly influences sensor response stability, temperature dependence, and application. A comparison of these three conduction types is provided in Table 2. Table 1 provides a quantitative comparison of these mechanisms where such metrics are available. A discussion of standardization and benchmarking protocols is provided in Section 3.8, and the proposed minimum reporting checklist is summarized in Table 3. Signal processing and readout electronics are discussed in Section 3.9. A comprehensive comparison of these mechanisms, including sensitivity, detection limit, response time, working range, stretchability, durability, power requirement, and typical applications, is provided in Table 4.

**Table 1:** Quantitative comparison of representative sensing mechanisms for hydrogel-based wearable sensors.

Mechanism	Sensitivity or Metric	Detection Range and Response/Recovery Time	Power Consumption	References
Piezoresistive	Ionic gauge factor typically 0.5 to 5; electronic composite gauge factor can exceed several hundred with controlled microcracks; unpatterned composite gels typically 1 to 10	Detection range up to 2800 percent strain for MXene-hydrogel heterostructure; response approximately 90 milliseconds and recovery approximately 240 milliseconds for MXene-hydrogel heterostructure	Low; compatible with constant-current source and voltmeter readout	[64]
Capacitive	Modest gauge factor; geometric capacitance change described by C equals epsilon A divided by d	Detection range includes pressure and strain; microstructured dielectric improves effective nonlinearity; response and recovery range from milliseconds to seconds depending on device geometry	Very low; capacitive readout draws essentially no direct current	[65]
Triboelectric	Open-circuit voltage and short-circuit current; output depends on surface charge density, contact area, and separation distance	Detection range includes contact-separation, lateral sliding, single-electrode, and freestanding triboelectric-layer modes; response time is in the millisecond range	Self-powered; microwatt-scale harvest budget	[66]
Ionic thermoelectric	Thermopower of 24.17 millivolts per kelvin	Detection range includes temperature gradients and spatial thermal fields; response time is seconds to reach steady state	Self-powered; low-grade thermal energy harvesting	[67]
Electrochemical	Faradaic current or Nernstian potential scales with analyte concentration	Detection range from micromolar to nanomolar concentrations for metabolites, drugs, and proteins; response time is minutes	Low; requires potentiostat and stable reference electrode	[68]
Multimodal decoupling	Decoupling accuracy and crosstalk floor rather than a single sensitivity metric	Detection range includes multiple stimuli such as strain, temperature, humidity, and pressure; response time depends on stack architecture and algorithm	Depends on readout electronics and processing	[69]

**Table 2:** Comparison of ionic, electronic, and mixed conduction in hydrogel sensors.

Conduction Type	Charge Carriers and Transport Mechanism	Conductivity, Temperature Dependence, and Response Stability	Advantages, Limitations, and Typical Applications	References
Ionic conduction	Mobile ions such as Na^+^, Li^+^, Ca^2+^, and Cl^−^ migrate through the aqueous phase; transport is governed by ion concentration, mobility, and polymer network structure	Conductivity tunable over orders of magnitude; strong temperature dependence because ion mobility depends on temperature; response stability is moderate and sensitive to humidity, dehydration, and freezing	Advantages: tissue-like compliance, biocompatibility, low impedance, and compatibility with biological signaling. Limitations: dehydration, freezing, ion migration, and electrode polarization. Typical applications: piezoresistive, capacitive, electrochemical, and ionic thermoelectric sensors	[7,8,9,10]
Electronic conduction	Electrons and holes transport through percolated conductive fillers such as carbon nanotubes, graphene, MXene, and liquid metals; transport is governed by filler network connectivity and tunneling gaps	Conductivity high and dependent on percolation; weak to moderate temperature dependence; response stability is high and less sensitive to humidity than ionic conduction	Advantages: high sensitivity, fast response, and good stability. Limitations: rigid fillers can reduce stretchability, and percolation networks can be unstable under repeated deformation. Typical applications: piezoresistive, capacitive, and triboelectric sensors	[13,14,15]
Mixed conduction	Both mobile ions and electronic carriers contribute to charge transport; ionic and electronic pathways coexist in the same network	Conductivity broad and tunable by filler loading and ion concentration; temperature dependence is complex because it depends on both ionic and electronic pathways; response stability is high if the two pathways are properly decoupled	Advantages: combines high sensitivity with tissue-like compliance and can support multimodal sensing. Limitations: crosstalk, complex calibration, and material compatibility issues. Typical applications: multimodal sensing, hybrid piezoresistive and electrochemical devices, and intelligent wearables	[22,69]

### 3.1. Piezoresistive Sensing Characterization

Piezoresistive transduction converts mechanical deformation into resistance change and remains the most widely implemented mechanism in hydrogel sensors, owing to its simplicity of readout (a constant current source and a voltmeter suffice), its compatibility with both ionic and electronic conduction pathways, and the maturity of its design rules. The resistance response of a composite hydrogel originates from two coupled processes. The first is geometric: stretching elongates the conduction path and reduces cross-sectional area, as the elementary relation R = ρL/A dictates; for incompressible networks this geometric contribution alone yields a modest, strain-linear response. The second is percolative: deformation disrupts the connectivity of conductive fillers, breaking conducting pathways and widening inter-filler tunneling gaps. Because tunneling current decays exponentially with gap distance—typically an order of magnitude of current change per ångström-scale gap widening—modest structural changes produce dramatically amplified resistance changes. This exponential sensitivity is the fundamental advantage of the mechanism, and virtually every high-gain piezoresistive hydrogel exploits it.

It is worth distinguishing the two conduction regimes that coexist in composite gels. In ionically conductive networks, current is carried by hydrated ions migrating through the aqueous phase; strain changes resistance mainly through geometric and ion-concentration effects, and the response is linear but modest (GF typically 0.5–5). In electronically conductive composites—CNT, graphene, MXene, liquid metal fillers—current crosses filler–filler junctions by tunneling, and the response can be enormous but nonlinear. The highest-performance devices deliberately hybridize the regimes: an ionic background conduction provides a stable baseline and self-healing capability, while a sparse electronic percolation network supplies the exponential amplification.

Micropatterned composite hydrogel sheets with surface electronic conductive networks exemplify a representative advanced design (Figure 5A) [64]. In these devices, CNT-embedded surface micropatterns concentrate stress at deliberately engineered locations, generating controlled microcracks whose propagation under load and recovery upon unloading produce large, repeatable resistance modulation. The crack-based amplification strategy can yield gauge factors (GF = (ΔR/R_0_)/ε) exceeding several hundred at small strains, whereas unpatterned composite gels typically deliver GF values of 1–10. The price of this amplification is well documented: controlled cracking narrows the linear range, introduces hysteresis from imperfect crack closure, and accelerates fatigue because each cycle re-damages the same defect sites [19,65,70,71,72,73]. Design refinements—gradient filler distribution that stages crack activation across strain levels, crack-tip blunting via elastomeric interlayers that arrest propagation, and self-similar hierarchical patterning that distributes damage spatially—continue to push the sensitivity–durability frontier, but the fundamental trade-off between signal amplification and mechanical longevity remains the defining constraint of piezoresistive hydrogels. For quantitative comparison, the key metrics of piezoresistive hydrogel sensors include gauge factor, strain range, response time, recovery time, hysteresis, and cyclic stability. Ionic hydrogels typically exhibit gauge factors between 0.5 and 5, whereas electronically conductive composites with controlled microcracks can exceed several hundred at small strains. Unpatterned composite gels typically deliver gauge factors between 1 and 10. The MXene-hydrogel heterostructure achieves a working range of 2800 percent, a response time of approximately 90 milliseconds, and a recovery time of approximately 240 milliseconds. These values are summarized in Table 1.

### 3.2. Capacitive Sensing Dynamics

Capacitive transduction encodes deformation as capacitance change, C = εA/d, where ε is the dielectric permittivity, A the electrode overlap area, and d the electrode separation. In the canonical parallel-plate configuration, compressing a hydrogel dielectric reduces d and increases C; stretching increases area while thinning the dielectric, producing a combined response that can be engineered to be either sensitive to or, usefully, insensitive to strain. A flexible capacitive pressure sensor based on 3D porous ionogel demonstrates the architectural sophistication now achievable: compressible microstructured dielectric layers amplify the geometric response to pressure, so that small normal loads produce large, well-resolved capacitance shifts for wearable health monitoring (Figure 5B) [65].

Compared with their piezoresistive counterparts, capacitive hydrogel sensors offer three systematic advantages. First, power consumption is lower because capacitive readout draws essentially no DC current; this matters for continuous monitoring applications where battery life dominates user experience and for implanted devices where battery replacement is impossible. Second, hysteresis is typically smaller because capacitance depends on geometry rather than on defect evolution—no microcracks need to open and close, and no percolation network needs to re-form; the measurement is therefore highly repeatable across millions of cycles. Third, the measurement is insensitive to contact resistance fluctuations at the electrode interface, a chronic noise source in resistive devices worn on skin that perspires and whose electrode contact quality varies with motion.

The corresponding limitations are equally clear. Parasitic capacitance from nearby conductors—including the human body itself, which is a conductive mass at typical measurement frequencies—corrupts the signal and demands guarded electrode layouts and differential readout. The readout circuitry is more complex than a simple voltage divider, typically requiring capacitance-to-digital conversion with femtofarad resolution. The achievable gauge factors are generally modest, since geometric capacitance changes linearly with strain, though microstructured dielectrics with pore-collapsing architectures substantially improve the effective nonlinearity. Finally, the classic parasitic sensitivity to humidity—since the dielectric permittivity of the hydrated network changes with water content—requires either encapsulation or compensation [19,70,71]. Consequently, capacitive architectures dominate where stability and power efficiency outweigh raw sensitivity—chronic health monitoring, implantable telemetry, and large-area arrays—while piezoresistive architectures retain the high-sensitivity niche. For quantitative comparison, the key metrics of capacitive hydrogel sensors include normalized capacitance change, pressure or strain range, response time, recovery time, power consumption, hysteresis, and humidity sensitivity. Capacitive sensors typically exhibit modest gauge factors because geometric capacitance changes linearly with strain, but microstructured dielectrics with pore-collapsing architectures substantially improve the effective nonlinearity. Power consumption is very low because capacitive readout draws essentially no direct current. These values are summarized in Table 1.

### 3.3. Triboelectric and Contact Electrification Mechanisms

Triboelectric nanogenerators (TENGs) harvest the contact electrification that occurs whenever two materials of differing electron affinity touch and separate, converting ambient mechanical cycles into electrical output without any external power source. In hydrogel implementations, the ionic gel typically serves as the compliant, stretchable electrode while an elastomeric counterpart (silicone, PTFE, FEP) acts as the tribo-negative layer; cyclic contact induces charge transfer that the hydrogel electrode collects, producing an open-circuit voltage and short-circuit current that encode the contact dynamics. The physics is deceptively simple—surface charge density, contact area, and separation distance jointly set the output—yet the design space it opens is vast.

Four canonical TENG modes have all been realized in hydrogel formats, each suited to different mechanical inputs. Vertical contact-separation mode captures normal pressing and releases and is the natural choice for tactile skins; lateral sliding mode converts tangential motion and suits trackpads and gesture surfaces; single-electrode mode simplifies wiring for wearables where the counter-surface is uncontrolled (floors, garments, grasped objects); and freestanding triboelectric-layer mode enables non-contact proximity sensing by coupling an independently moving charged object to patterned electrodes. Micro-pyramid patterned ionic hydrogel tactile sensors illustrate the mechanism’s deployment in human–machine interfaces (Figure 5C) [66]. Operating in dual-electrode and single-electrode modes, the patterned interface amplifies both the effective contact area modulation and the air-gap capacitance change during touch, achieving ultra-sensitive, deformable, and transparent tactile detection.

The self-powered nature of TENG sensing is a notable advantage: distributed sensing nodes on garments, prosthetics, and robotic skins can operate without batteries or wired power, and the output amplitude itself carries tactile information (contact force, area, and dynamics) without requiring a separate excitation source [67,74,75,76]. Power management circuits can rectify and buffer the harvested charge to operate intermittent wireless transmitters, though microwatt-scale harvest budgets confine these to low-duty-cycle telemetry. The limitations are environmental and electrical. Humidity degrades triboelectric charge retention, particularly problematic for hydrogels whose surfaces are inherently hydrated; surface hydrophobization or encapsulation layers mitigate this at some cost to skin conformity. Output impedance is high, demanding impedance-buffered readout. The AC output waveform requires rectification and conditioning before integration with digital logic. Hybrid designs that pair TENG harvesting with supercapacitive energy storage and near-field communication are progressively closing the gap between laboratory demonstration and deployable self-powered sensing. For quantitative comparison, the key metrics of triboelectric hydrogel sensors include open-circuit voltage, short-circuit current, surface charge density, response time, power output, and impedance. Triboelectric sensors are self-powered and can operate without batteries, but their output is alternating current and requires rectification and conditioning. Humidity degrades triboelectric charge retention, and the high output impedance demands impedance-buffered readout. These values are summarized in Table 1.

### 3.4. Ionic Thermoelectric Mechanisms

Ionic thermoelectric (iTE) sensing exploits the Soret effect: under an applied temperature gradient, mobile ions in a gel redistribute preferentially, with cations and anions migrating at different rates because their solvation shells and matrix interactions differ, so that a concentration gradient—and hence a thermovoltage—builds across the thickness of the material. Because ionic thermopowers reach tens of millivolts per kelvin, exceeding electronic Seebeck coefficients (typically tens of microvolts per kelvin) by three orders of magnitude, iTE hydrogels constitute a highly sensitive route to temperature sensing that simultaneously functions as a low-grade thermal energy harvester. The distinction between the ionic and electronic mechanisms is fundamental: electronic thermoelectrics convert heat flow to electricity via carrier energy filtering, whereas ionic systems build an actual concentration cell—the thermovoltage is a Nernst potential of the redistributed ions, and it persists as long as the gradient is maintained, until back-diffusion equilibrates the fluxes.

Ultrasensitive flexible thermal sensor arrays patterned from polyquaternium-10/NaOH ionic thermoelectric hydrogels demonstrate the application frontier: the giant thermopower of 24.17 mV K^−1^ achieved by this system endows prosthetic hands with thermal sensation, restoring a sensory modality that conventional prosthetics lack entirely (Figure 5D) [67]. The sensing mechanism is intrinsically multimodal in a useful sense: because the ionic Seebeck effect responds to temperature gradients rather than absolute temperature, iTE arrays map spatial thermal fields—the direction and intensity of heat sources—which point sensors cannot do. A prosthetic fingertip carrying an iTE array can localize a warm object before contact.

The principal challenges are response speed and signal stability. Ion redistribution over millimeter scale distances requires seconds to reach steady state, limiting the bandwidth of thermal tracking; and the concentration gradient relaxes under sustained steady gradients, causing the thermovoltage to drift from its peak toward an equilibrium value. Pulsed measurement protocols that sample the initial fast response, and materials engineering that stabilizes ion–matrix interactions to widen the relaxation time, have substantially mitigated both limitations [74,75,76]. Combined with the fact that body-adjacent temperature gradients are ubiquitous (skin–air, skin–garment, distal–proximal vasculature), iTE hydrogels are emerging as a common choice for passive, self-powered thermal mapping in wearable systems. For quantitative comparison, the key metrics of ionic thermoelectric hydrogel sensors include thermopower, response time, stability under sustained temperature gradients, and power output. The polyquaternium-10/NaOH system achieves a thermopower of 24.17 millivolts per kelvin, which is more than an order of magnitude above electronic Seebeck coefficients. However, ion redistribution over millimeter-scale distances requires seconds to reach steady state, and the thermovoltage drifts under sustained steady gradients. These values are summarized in Table 1.

### 3.5. Electrochemical Recognition in Gel Networks

Electrochemical transduction converts molecular recognition into measurable current or potential, extending hydrogel sensing from mechanical quantities to the chemical composition of biofluids. Three sub-modes dominate. Amperometric sensing drives an analyte through redox chemistry at a biased electrode surface; the resulting faradaic current scales with analyte concentration and underlies enzyme-based glucose and lactate detection. Potentiometric sensing measures the Nernstian potential developed across an ion-selective membrane; it is the workhorse of pH, Na^+^, K^+^, and ammonium sweat analysis. Impedimetric sensing tracks the interfacial impedance spectrum, whose changes report binding events at modified electrodes; it powers aptamer and immunosensor formats. Hydrogels contribute uniquely to all three: they confine aqueous electrolytes against the skin, host immobilized enzymes and aptamers at high loading in their hydrated meshes, and swell to admit analyte while excluding interferents by size and charge.

Integrated electrochemical aptamer biosensing platforms represent the state of the art (Figure 5E) [68]. Structure-switching aptamer probes grafted onto flexible electrodes undergo conformational rearrangement upon target binding, producing a measurable faradaic current shift that continuously tracks drug pharmacokinetics in interstitial fluid; hydrogel microneedle arrays sample that fluid painlessly, while phenol-red-loaded microneedle patches report infection-site pH through visible color shifts readable by smartphone camera. The strength of electrochemical sensing is molecular specificity: enzymatic recognition (glucose oxidase, lactate oxidase, alcohol oxidase) and aptamer recognition (vancomycin, gentamicin, cytokines) extend detection to metabolites, drugs, and proteins at micromolar to nanomolar concentrations that no physical mechanism can reach [77,78]. Its weaknesses are calibration drift from enzyme deactivation and electrode fouling, dependence on stable reference electrodes—a chronic difficulty in wearable formats where the conventional Ag/AgCl junction dries out—and sample access, since biofluid must be reliably delivered to the sensing surface, a constraint that motivates the microfluidic and osmotic collection strategies reviewed in Section 6 [69]. For quantitative comparison, the key metrics of electrochemical hydrogel sensors include detection limit, sensitivity, response time, calibration stability, and selectivity. Electrochemical sensing uniquely reaches molecular analytes at micromolar to nanomolar concentrations, but its performance is limited by calibration drift from enzyme deactivation and electrode fouling, dependence on stable reference electrodes, and sample access. These values are summarized in Table 1.

### 3.6. Multimodal Decoupling and Mechanism Modeling

The gravest untapped difficulty in hydrogel sensing is crosstalk: ionic conductivity is simultaneously sensitive to strain, temperature, humidity, and pressure, so a single-channel resistance measurement cannot attribute its change to any one stimulus. A sensor worn on a sweating forearm during exercise registers deformation, thermal gradient, and moisture flux simultaneously; without decoupling, the signal is ambiguous, and every downstream interpretation inherits the ambiguity. Multimodal decoupling strategies attack this problem through three complementary layers of co-design.

Materials-level decoupling engineers orthogonal sensitivities into different network components: a temperature-insensitive ionic channel paired with a temperature-sensitive thermochromic phase, or a humidity-buffered organohydrogel matrix that removes one variable from the problem entirely. Device-level decoupling exploits geometry and stack architecture. Hydrogel stack-tailored logic devices demonstrate the most physically elegant approach (Figure 5F) [69]: by laminating hydrogel layers with engineered, spatially distinct ion distributions, the device exploits the fact that different stimuli drive ion migration along different spatial coordinates; evaluating the ionic migration with precision allows each stimulus component to be reconstructed from the composite signal, achieving high-fidelity multimodal sensing through the stack architecture alone. Algorithm-level decoupling completes the toolkit: differential sensor pairs with deliberately mismatched stimulus sensitivities can be ratioed to cancel the common mode, and machine-learning regression can learn the crosstalk manifold from training data and invert it at inference time. The stack approach carries the advantage of interpretability—its physics is transparent and calibration-free—while learned approaches offer flexibility when the crosstalk is too complex to model. The field is converging on hybrid solutions in which physically engineered decoupling provides the scaffolding and lightweight algorithms polish the residual errors. For quantitative comparison, the key metrics of multimodal decoupling include decoupling accuracy, crosstalk floor, residual error after decoupling, response time, and stability over time. The hydrogel stack-tailored logic device achieves high-fidelity multimodal sensing by evaluating ionic migration with precision, but the crosstalk manifold drifts as the sensor ages. These values are summarized in Table 1.

**Table 3:** Minimum reporting checklist for hydrogel sensor benchmarking.

Category	Parameter	Recommended Reporting	Rationale
Sensitivity	Gauge factor or normalized signal change	Definition of gauge factor, strain or pressure range, linear range, coefficient of determination	Enables comparison of sensitivity across studies
Response	Response time and recovery time	Explicit definition, such as time to reach 90 percent of steady-state value or time to reach 63 percent of steady-state value	Avoids ambiguity in reported speed
Hysteresis	Hysteresis and cyclic stability	Maximum hysteresis normalized to full-scale output, number of cycles, strain or pressure amplitude, frequency	Quantifies repeatability and fatigue
Environment	Temperature, humidity, encapsulation	Test temperature, relative humidity, packaging or encapsulation details	Enables assessment of real-world stability
Self-healing	Mechanical healing efficiency and electrical healing efficiency	Healing time, healing conditions, recovered fracture strength or toughness, recovered conductivity	Enables comparison of healing performance
Adhesion	Adhesion strength	Peel test, lap shear test, or probe tack test, substrate, surface conditions	Important for skin-contact applications
Biocompatibility	ISO 10993 tests	ISO 10993-1 [79], ISO 10993-5 [80], ISO 10993-10 [81], ISO 10993-11 [82], leachables and degradation products	Ensures safety for wearable and implantable use
Long-term stability	Continuous wear and aging	Weeks to months of continuous-wear data, accelerated aging data, calibration drift data, storage conditions	Assesses operational lifetime
Multimodal crosstalk	Cross-sensitivity matrix	Decoupling method, residual error after decoupling, cross-sensitivity coefficients	Enables reliable multimodal sensing
Statistics	Sample size and variability	Number of devices tested, mean, standard deviation, statistical method, batch-to-batch variability	Improves reproducibility

### 3.7. Comparative Assessment

Table 1 summarizes the quantitative comparison of the six representative mechanisms where such metrics are available. The table includes sensitivity or gauge factor, detection range, response and recovery time, power consumption, linearity and hysteresis, advantages, limitations, and representative references. Quantitatively, the comparison shows that piezoresistive sensors offer the highest sensitivity per unit complexity but suffer from nonlinearity and fatigue. Capacitive sensors offer lower power consumption and lower hysteresis but have modest gauge factors and are sensitive to parasitic capacitance and humidity. Triboelectric sensors are self-powered and can operate without batteries, but their output is alternating current and requires conditioning. Ionic thermoelectric sensors achieve thermopowers above 20 millivolts per kelvin but respond slowly and drift under sustained gradients. Electrochemical sensors specifically reach molecular analytes at micromolar to nanomolar concentrations but require stable reference electrodes and suffer from calibration drift. Multimodal decoupling does not have a single sensitivity metric; instead, it is evaluated by decoupling accuracy and crosstalk floor, which remain difficult to quantify consistently across studies. These quantitative differences are summarized in Table 1. A broader comparison that includes working range, stretchability, durability, and typical applications is provided in Table 4. However, cross-study comparison of these mechanisms remains difficult because reporting protocols are not standardized. Different studies use different definitions of gauge factor, different strain ranges, different response and recovery time criteria, and different test environments. Section 3.8 therefore proposes a minimum reporting checklist for hydrogel sensor benchmarking, which is summarized in Table 3. Signal processing and readout electronics for these mechanisms are discussed in Section 3.9.

### 3.8. Standardization and Benchmarking Protocols for Hydrogel Sensors

Cross-study comparison of hydrogel sensors is currently limited by inconsistent reporting practices. Studies often define sensitivity differently, use different strain or pressure ranges, report response and recovery times under different criteria, and test under different temperature and humidity conditions. To enable meaningful benchmarking, we propose a minimum reporting checklist for future hydrogel sensor studies. Table 3 summarizes this checklist, and the main items are explained below.

First, studies should clearly define the gauge factor and the strain or pressure range over which it is measured. For resistive sensors, the gauge factor should be reported as the slope of relative resistance change versus strain, together with the linear range and the coefficient of determination. For capacitive sensors, the normalized capacitance change should be reported together with the electrode geometry and dielectric thickness.

Second, response and recovery times should be defined explicitly. The definition should state whether the response time is the time to reach 90 percent of the steady-state value, the time to reach 63 percent of the steady-state value, or another criterion. The same definition should be applied consistently across all measurements.

Third, hysteresis should be quantified. Hysteresis can be reported as the maximum difference between loading and unloading curves at the same stimulus level, normalized to the full-scale output. Cyclic stability should be reported with the number of cycles, the strain or pressure amplitude, and the frequency.

Fourth, environmental conditions should be reported. Temperature, relative humidity, and any encapsulation or packaging should be specified. For wearable applications, studies should report performance after sweating, bending, and prolonged skin contact where possible.

Fifth, self-healing performance should be reported using both mechanical healing efficiency and electrical healing efficiency. Mechanical healing efficiency can be defined as the ratio of healed fracture strength or toughness to pristine values. Electrical healing efficiency can be defined as the ratio of healed conductivity to pristine conductivity. The healing time and healing conditions should also be reported.

Sixth, adhesion strength should be reported for skin-contact applications. Adhesion can be measured by peel tests, lap shear tests, or probe tack tests, and the substrate and surface conditions should be specified.

Seventh, biocompatibility should be evaluated according to ISO 10993 standards where skin contact or implantation is intended. Relevant tests include ISO 10993-1 for biological evaluation, ISO 10993-5 for in vitro cytotoxicity, ISO 10993-10 for sensitization and irritation, and ISO 10993-11 for systemic toxicity [79,80,81,82]. Leachables and degradation products should be characterized.

Eighth, long-term stability should be reported. Studies should provide continuous-wear data over weeks or months, accelerated aging data, and calibration drift data. The storage conditions and encapsulation strategy should be described.

Ninth, multimodal crosstalk should be quantified. When a sensor responds to more than one stimulus, the ISO 10993-6 [83] cross-sensitivity matrixould be reported. The decoupling method, whether materials-level, device-level, or algorithm-level, should be described, and the residual error after decoupling should be quantified.

Tenth, statistical reporting should be improved. Studies should report sample size, number of devices tested, mean and standard deviation, and the statistical method used. Batch-to-batch variability should be reported for at least three independent fabrication batches.

Adopting this minimum reporting checklist would greatly improve reproducibility and enable meaningful comparison across hydrogel sensor platforms.

### 3.9. Signal Processing and Readout Electronics

Practical wearable deployment requires readout electronics matched to each transduction mechanism. Resistive sensors typically use constant-current or voltage-divider readout, but AC impedance measurement is preferred to avoid electrode polarization. Capacitive sensors require capacitance-to-digital conversion with femtofarad resolution and guarded electrode layouts to suppress parasitic capacitance. Triboelectric sensors generate high-impedance AC outputs and therefore need charge amplifiers or impedance-buffered readout, followed by rectification. Ionic thermoelectric sensors produce small DC voltages and require low-noise amplification and drift compensation. Electrochemical sensors rely on potentiostats and stable reference electrodes. Across all modalities, filtering, shielding, common-mode rejection, and drift compensation are essential. Wireless telemetry via Bluetooth Low Energy or near-field communication enables wearable operation, while machine-learning-assisted signal processing can mitigate crosstalk and nonstationarity. The choice of readout architecture also affects power consumption, form factor, and cost. For continuous monitoring, low-power analog front ends and duty-cycled operation are necessary. For multimodal sensing, synchronized sampling across channels and real-time decoupling algorithms are required. For self-powered sensors, energy harvesting and power management circuits must be co-designed with the sensing mechanism. Therefore, signal processing and readout electronics should be treated as an integral part of hydrogel sensor design rather than as an afterthought.

**Table 4:** Comprehensive comparison of sensing mechanisms for hydrogel-based wearable sensors.

Mechanism	Sensitivity and Detection Limit	Response Time, Working Range, and Stretchability	Durability, Power Requirement, and Typical Applications	References
Piezoresistive	Ionic gauge factor typically 0.5 to 5; electronic composite gauge factor can exceed several hundred with controlled microcracks; strain detection down to small strains; pressure detection depends on microstructure	Response approximately 90 milliseconds and recovery approximately 240 milliseconds for MXene-hydrogel heterostructure; working range up to 2800 percent strain; high stretchability	Moderate durability; fatigue from repeated cracking and hysteresis; low power requirement and compatible with constant-current source and voltmeter readout; typical applications include motion monitoring, pressure sensing, electronic skin, and consumer wearables	[64]
Capacitive	Modest gauge factor; normalized capacitance change; pressure and strain detection; femtofarad-level capacitance resolution required	Response and recovery from milliseconds to seconds depending on device geometry; working range includes pressure and strain; high stretchability	Good durability; low hysteresis and high repeatability across millions of cycles; very low power requirement because capacitive readout draws essentially no direct current; typical applications include chronic health monitoring, implantable telemetry, large-area arrays, and sleep tracking	[65]
Triboelectric	Open-circuit voltage and short-circuit current; output depends on surface charge density, contact area, and separation distance; contact and separation detection; tactile force and area	Response time in the millisecond range; working range includes contact-separation, lateral sliding, single-electrode, and freestanding modes; high stretchability	Moderate durability; humidity degrades charge retention and high output impedance requires conditioning; self-powered with microwatt-scale harvest budget; typical applications include tactile sensing, self-powered interfaces, robotic skins, and wearables	[66]
Ionic thermoelectric	Thermopower of 24.17 millivolts per kelvin; temperature gradient detection; spatial thermal fields	Response time is seconds to reach steady state; working range includes temperature gradients and spatial thermal fields; high stretchability	Moderate durability; drift under sustained gradients; self-powered and based on low-grade thermal energy harvesting; typical applications include thermal mapping, prosthetic thermal sensation, and body-adjacent temperature monitoring	[67]
Electrochemical	Faradaic current or Nernstian potential scales with analyte concentration; micromolar to nanomolar detection for metabolites, drugs, and proteins	Response time is minutes; working range covers molecular analytes in biofluids; moderate stretchability	Moderate durability; calibration drift from enzyme deactivation and electrode fouling; low power requirement but requires potentiostat and stable reference electrode; typical applications include sweat sensing, interstitial diagnostics, drug monitoring, and wound infection detection	[68]
Multimodal decoupling	Decoupling accuracy and crosstalk floor rather than a single sensitivity metric; multiple stimuli including strain, temperature, humidity, and pressure	Response time depends on stack architecture and algorithm; working range covers multi-stimulus sensing; high stretchability	Moderate durability; crosstalk manifold drifts as sensor ages; power requirement depends on readout electronics and processing; typical applications include multimodal sensing, human–machine interaction, and intelligent wearables	[69]

## 4. Performance Optimization and Structural Characterization

**Figure 6:**
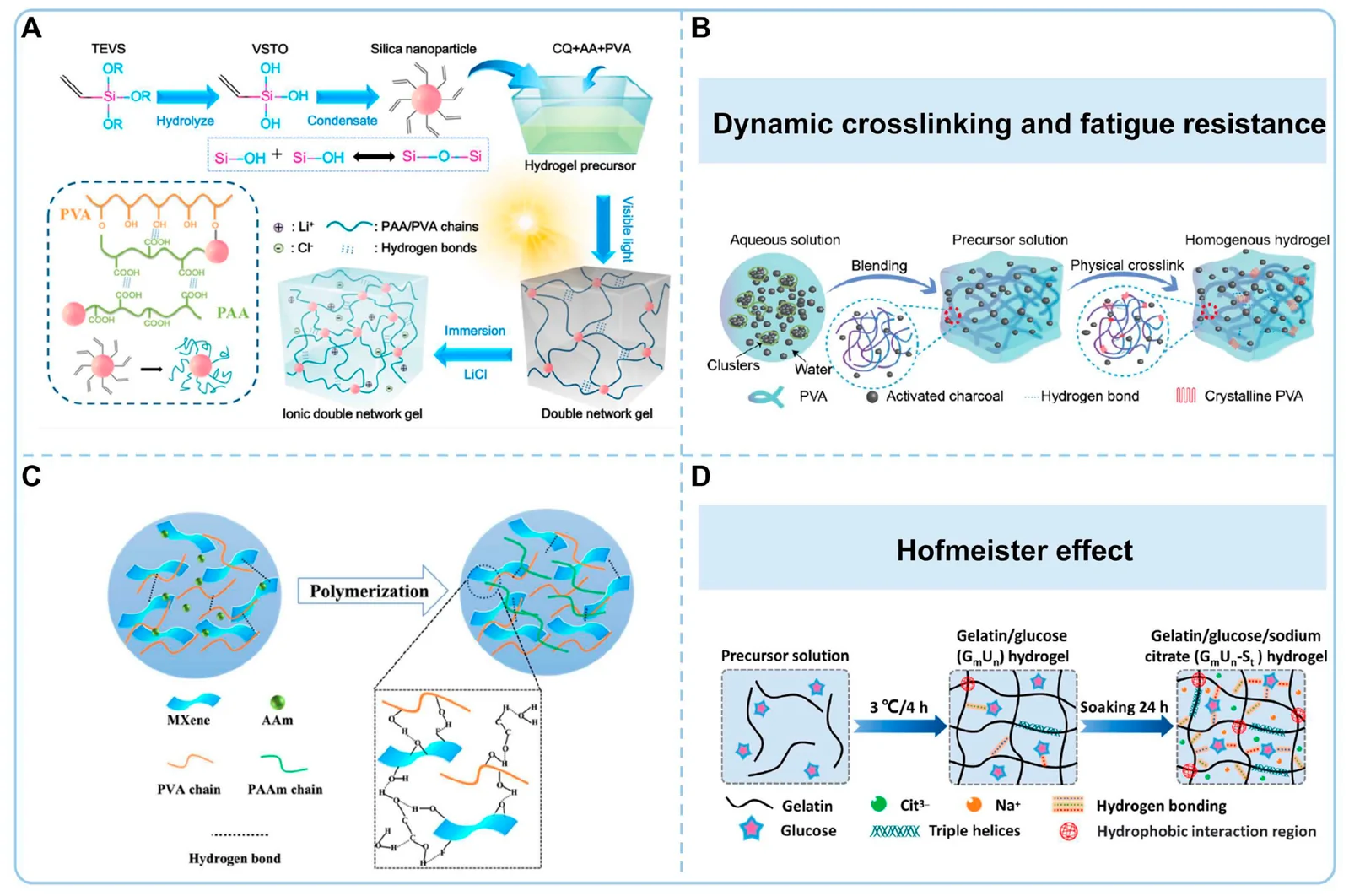
Mechanical enhancement strategies of hydrogel sensors: (**A**) Energy dissipation of double network with multiple sacrificial bonds, integrating silica nanoparticle sol–gel network and covalent PAA/PVA interpenetrating structure. Reproduced with permission from Ref. [84]. Copyright *Polymers*. (**B**) Anti-fatigue design via physical crosslinking, based on PVA crystallites formed by freeze–thaw cycles and conductivity reinforcement of activated carbon. Reproduced with permission from Ref. [87]. Copyright *Polymers*. (**C**) Nanofiller reinforcement, achieving dry-resistant human motion monitoring based on hydrogen-rich MXene/polymer interfaces. Reproduced with permission from Ref. [90]. Copyright *Polymers*. (**D**) Hofmeister effect engineering, where citrate-induced salting-out of gelatin/glucose network promotes triple-helix densification. Reproduced with permission from Ref. [94]. Copyright *Gels*.

**Figure 7:**
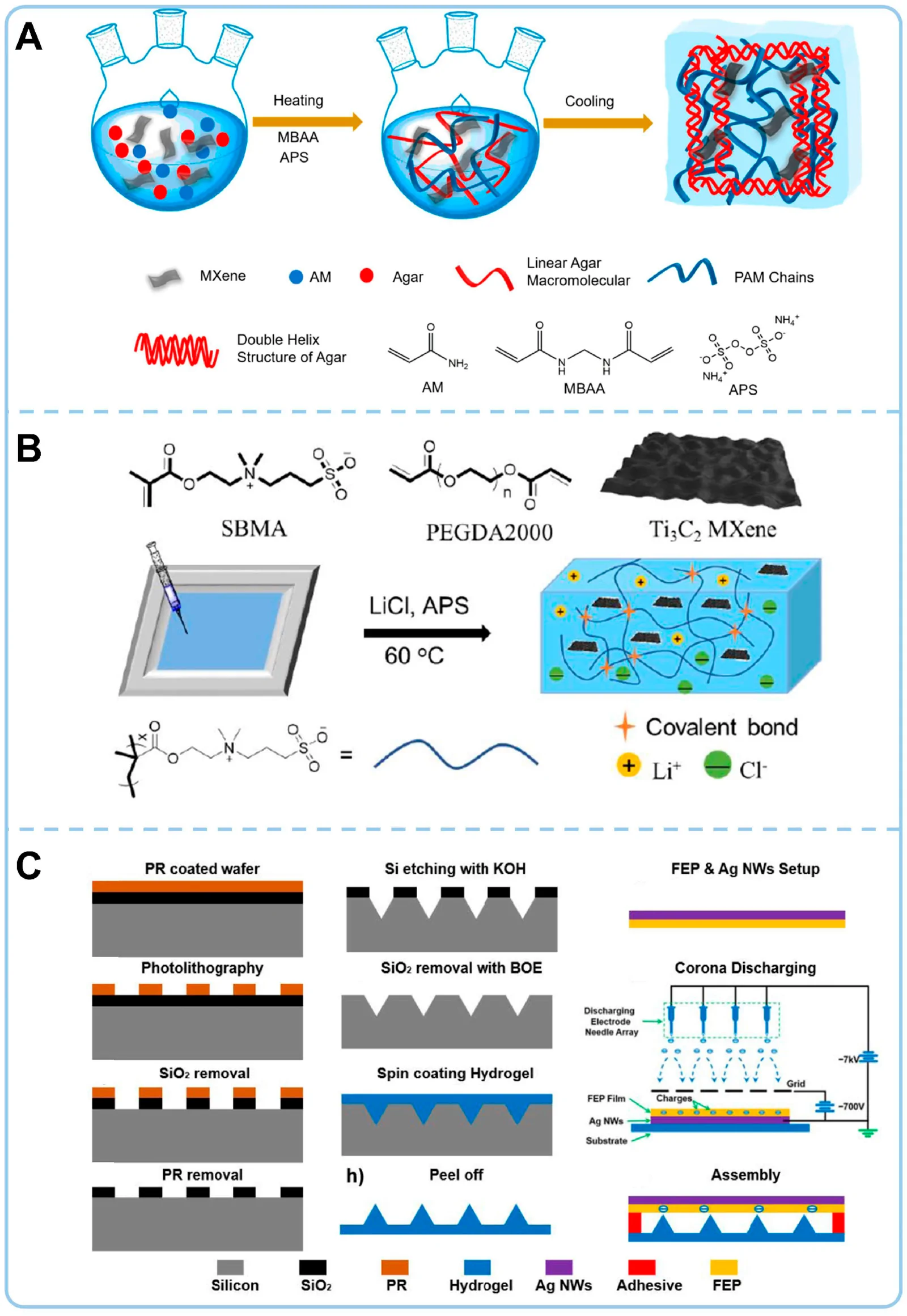
Sensitivity enhancement strategies of hydrogel sensors: (**A**) Low-temperature adaptable double-network MXene nanocomposite hydrogel combining thermoreversible agar/PAM network and MXene percolation network. Reproduced with permission from Ref. [97]. Copyright *Micromachines*. (**B**) Alkali-ion accelerated gelation for dense MXene networks to achieve sharper percolation response. Reproduced with permission from Ref. [98]. Copyright *Gels*. (**C**) Electret/hydrogel-based tactile sensor integrating charge-injected micropatterned pyramid layer for electrostatically enhanced tactile detection. Reproduced with permission from Ref. [102]. Copyright *Micromachines*.

Material architecture determines what a hydrogel sensor could theoretically achieve; performance enhancement strategies determine what it actually achieves in service. Two performance axes dominate the literature: mechanical robustness (strength, toughness, fatigue resistance, recoverability), which governs survival under the cyclic loading inherent to wearable deployment, and sensitivity (gauge factor, detection limit, signal-to-noise ratio), which governs the resolution of the quantities being sensed. The two axes are only weakly coupled in principle but strongly coupled in practice, since the microstructural features that amplify signals are frequently the same features that concentrate stress, and the strategies addressing them are accordingly treated separately before their coupling is examined (Figure 6 and Figure 7).

### 4.1. Mechanical Enhancement of Gel Networks

Double-network energy dissipation. As introduced in Section 1.2, mechanical toughness is governed by network architecture and energy dissipation. The DN concept introduced in Section 2.1 reaches its fullest development in multi-sacrificial-bond designs (Figure 6A) [84]. Multi-sacrificial-bond DN hydrogels integrate silica-nanoparticle sol–gel networks with covalently crosslinked PAA/PVA interpenetration, deploying an entire hierarchy of sacrificial interactions—ionic bonds, hydrogen bonds, and reversible covalent linkages—whose activation stresses span a wide range. Under deformation, the weakest bonds rupture first and dissipate energy at small strains, intermediate bonds engage as loading continues, and the covalent skeleton bears the residual stress; the sequential engagement yields high toughness, resilience, damping, and notch-insensitivity simultaneously. Theoretical treatments rationalize this behavior: Lake–Thomas-type models relate fracture energy to the number of chains bridging the crack plane, while molecular dynamics simulations reveal how network heterogeneity redirects crack-tip stress into surrounding sacrificial zones [85,86]. The practical consequence for sensing is twofold: devices survive the millions of deformation cycles typical of skin-mounted deployment—gait, respiration, pulse, and grasp each impose 10^4^–10^6^ cycles per day of use—and the stress–strain hysteresis that would otherwise corrupt calibration remains bounded and repeatable, because the same bond hierarchy exhausts in the same sequence every cycle.

Physical crosslinking and anti-fatigue design. Where covalent DN chemistry demands multi-step synthesis, physical crosslinking offers a solvent-free, biocompatible alternative: PVA crystallites formed by freeze–thaw cycling act as reversible junctions whose melting and reformation allow the network to anneal damage in situ. Low-hysteresis, fatigue-resistant PVA/activated charcoal hydrogels exploit exactly this principle (Figure 6B) [87]. Repeated freeze–thaw cycles grow PVA crystalline domains that serve both as load-bearing junctions and as anchors for the hydrogen-bonded amorphous network; the activated charcoal dispersed through the matrix provides percolated conductive pathways while simultaneously reinforcing the network through interfacial hydrogen bonding. The resulting gels sustain long-term stable monitoring—demonstrated through continuous plant growth tracking over weeks—with resistance drift below a few percent, a benchmark that few covalent systems match. Hydrogen-bond engineering between PVA hydroxyl groups and filler surfaces further stabilizes the network against creep under sustained load [88,89]. The anti-fatigue physics is instructive: because crystallite junctions are reversible, each load cycle remodels rather than accumulates damage, converting a fatigue problem into an annealing process—an inversion that explains why physically crosslinked systems frequently outlast chemically crosslinked ones in cyclic service despite lower single-cycle toughness.

Nanofiller reinforcement. Dispersed nanoscale fillers—MXene nanosheets, cellulose nanocrystals, silica nanoparticles, aramid nanofibers—reinforce hydrogels through interfacial load transfer, crack-pinning, and network bridging, provided the filler–matrix interface is properly engineered. Dry-resistant MXene nanocomposite conductive hydrogels for human motion monitoring demonstrate the payoff of interface-rich design (Figure 6C) [90]: hydrogen-bond-dense MXene/polymer interfaces maintain both mechanical flexibility and electrical percolation even after repeated dehydration–rehydration cycles, because the same interfacial hydrogen bonds that transfer stress also immobilize water against evaporation. Particulate fillers act as multifunctional junctions connecting multiple chains, while high-aspect-ratio fibrous fillers bridge microcracks and delay coalescence [91,92,93]. The design rules are now reasonably well established: filler loading must exceed the mechanical percolation threshold but stay below the agglomeration threshold, above which stress concentrates at agglomerates and toughness collapses; surface chemistry must maximize specific adhesion without immobilizing the surrounding chains, which would trade toughness for stiffness; and filler geometry should match the dominant failure mode—platelets against delamination, fibers against tearing, spheres against cavitation. When these rules are satisfied, reinforcement and conductivity arrive together, since the same percolated filler network serves both functions.

Hofmeister-effect engineering. The most recent addition to the mechanical toolbox uses ion-specific solvation to restructure the network from within. Mechanically robust and conductive gelatin/glucose hydrogels exploit citrate-mediated salting-out: immersion in sodium citrate at 3 °C followed by 24 h equilibration compacts the gelatin chains, promotes triple-helix formation, and densifies the network into a mechanically strong matrix suitable for flexible strain sensing (Figure 6D) [94]. The Hofmeister series ranks ions by their salting-out propensity; citrate and sulfate anions sit at the strongly kosmotropic end, withdrawing hydration water from the polymer and driving chain collapse [95,96]. The elegance of the approach lies in its operational simplicity—no new covalent chemistry, no filler processing, only a salt bath—yet the mechanical enhancement rivals filler reinforcement. Because the treatment also concentrates the ionic content of the gel as water is expelled, conductivity rises in parallel with strength, partially resolving the conductivity–mechanics dilemma identified in Section 1.2. Salting-out strategies are accordingly spreading rapidly through biomass-derived hydrogel systems where the starting polymers (gelatin, starch, cellulose derivatives) are intrinsically salting-out-responsive, and where the mild, aqueous processing preserves the biocompatibility that motivates these matrices in the first place.

### 4.2. Sensitivity Enhancement and Microstructural Characterization

Material-level strategies. As discussed in Section 3, sensitivity depends on the sensing mechanism and microstructure. This section focuses on strategies to enhance sensitivity and on the microstructural characterization of hydrogel sensors. Sensitivity begins with the conductive network architecture, and the deepest principle is that sensitivity lives at structural instabilities—the percolation threshold, the sol–gel transition, the onset of strain-induced localization—and the art is to park the sensor precisely at an instability without tipping it over. Low-temperature adaptive dual-network MXene nanocomposite hydrogels combine a thermoreversible agar/PAM network with MXene percolation, sustaining wearable strain sensing in cold environments while maintaining high gauge factors across the extended temperature window (Figure 7A) [97]. Alkali ion-accelerated gelation of MXene-based conductive hydrogels exploits OH^−^-triggered network densification not merely to shorten fabrication time but to sharpen the percolation threshold: faster gelation freezes the MXene dispersion into a finer, more interconnected network whose tunneling-gap statistics shift dramatically under strain, amplifying the resistance response (Figure 7B) [98]. Complementary material strategies tune ionic strength to place the sensor at the steepest point of its conductivity–concentration curve, adjust filler aspect ratio to maximize percolation sensitivity, and kinetically arrest network formation to preserve nonequilibrium microstructures whose rearrangement under load is large and rapid [99,100,101].

Structural engineering. Geometry multiplies material sensitivity, and it does so at negligible materials-development cost. An electret/hydrogel-based tactile sensor boosted by micropatterning and electrostatic promotion illustrates the multiplication vividly (Figure 7C) [102]: silicon-micromachined pyramidal templates transfer charge-injected FEP/Ag-NW layers onto patterned hydrogel films, so that each micro-pyramid concentrates contact stress at its apex while the embedded electret adds a bias field that amplifies the charge response to touch. The device achieves both the flexibility of the hydrogel and the wide-temperature tolerance of the electret, a combination neither component achieves alone. The broader toolkit of structural amplification shares a common logic: convert a spatially uniform deformation into a spatially concentrated event whose local signal change is large. Micropillars focus stress into small contact areas; wrinkled surfaces amplify area change geometrically as folds unfold; gradient porosity sequences pore collapse under compression, so that progressively stiffer strata engage as load increases, spreading high sensitivity across a wide pressure range [103,104,105,106]. Structural engineering composes freely with every material-level strategy, which is why the highest-performing reported sensors almost invariably combine both: a percolation-optimized composite microstructured into a stress-concentrating geometry.

### 4.3. Structural Integration: Coupling Mechanics and Sensitivity

The two enhancement families are conventionally reviewed separately but couple in practice through the network. Mechanical enhancement densifies and strengthens the network; densification shifts percolation and tunneling statistics, usually suppressing sensitivity; strengthening suppresses the microstructural rearrangements that high gauge factors exploit. Conversely, sensitivity engineering tends to embrittle—crack-based amplification is, by construction, damage-based amplification. The most successful recent designs acknowledge the coupling explicitly: sacrificial-bond networks provide toughness while their bond-exchange kinetics supply a recoverable sensitivity mechanism; crystallite junctions anneal fatigue damage while their melting transitions sharpen thermal sensitivity; Hofmeister compaction strengthens while simultaneously concentrating ions. Rather than maximizing a single axis, the next generation of enhancement strategies will likely be judged by the Pareto frontier they trace across mechanics, sensitivity, stability, and lifetime—and by how gracefully that frontier degrades under the combined stresses of real deployment, where temperature, humidity, and millions of deformation cycles act simultaneously.

## 5. Functional Properties: Self-Healing Chemistry and Gels in Mixed Solvents

**Figure 8:**
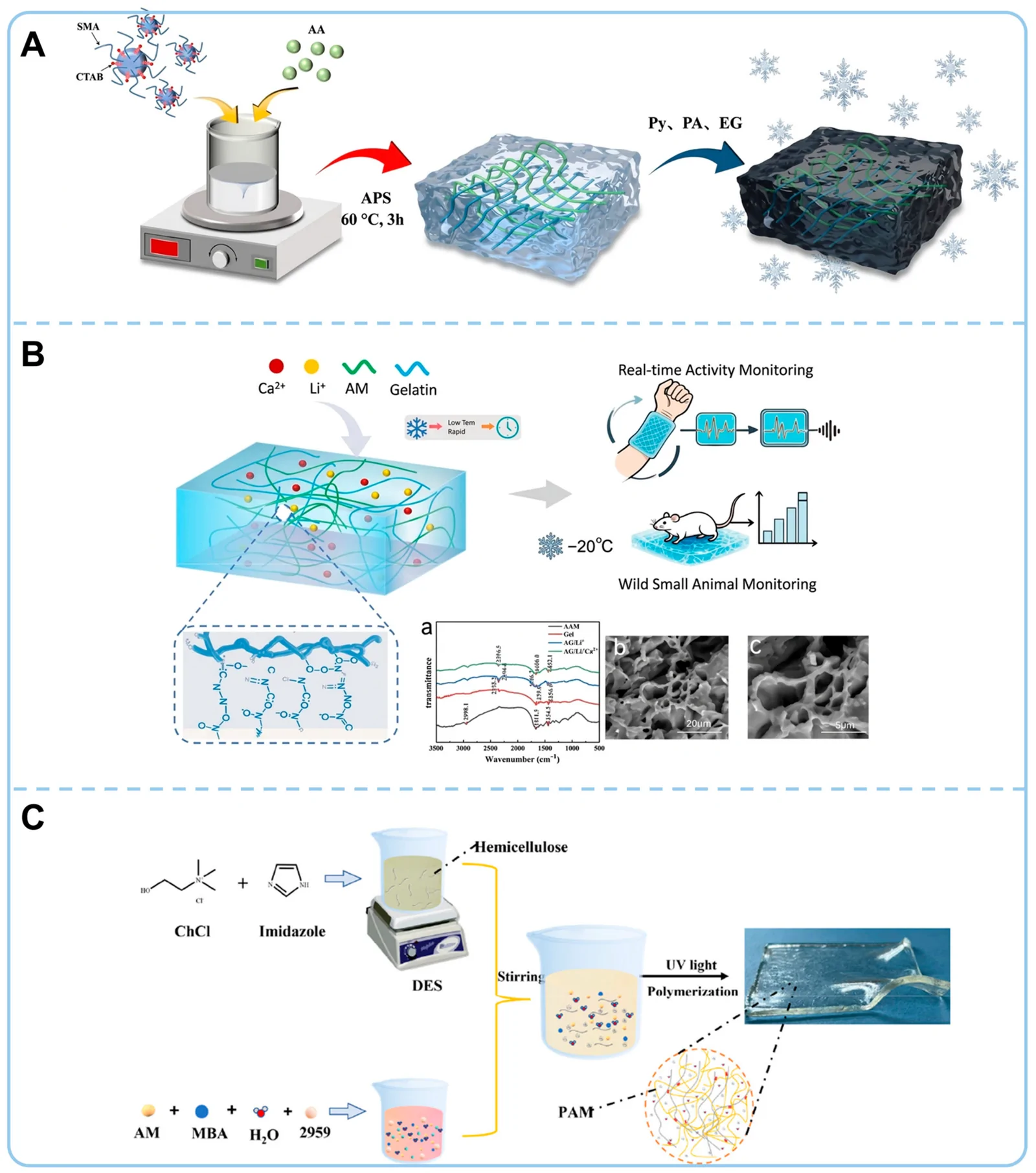
Anti-freezing and environmental stability strategies of hydrogel sensors: (**A**) Solvent replacement strategy combining hydrophobic association framework and dual antifreezing chemistry (freezing point depression of ethylene glycol and water confinement within hydrophobic domains). Reproduced with permission from Ref. [107]. Copyright *Polymers*. (**B**) Dual ionic salt incorporation strategy based on one-step Ca^2+^/Li^+^ ionically crosslinked acrylamide/gelatin network, capable of gelation at −20 °C. Reproduced with permission from Ref. [111]. Copyright *Sensors*. (**C**) Deep eutectic solvent engineering for antifreezing conductive biomass hydrogels fabricated by dissolving hemicellulose in choline chloride/imidazole. Reproduced with permission from Ref. [115]. Copyright *Gels*.

**Figure 9:**
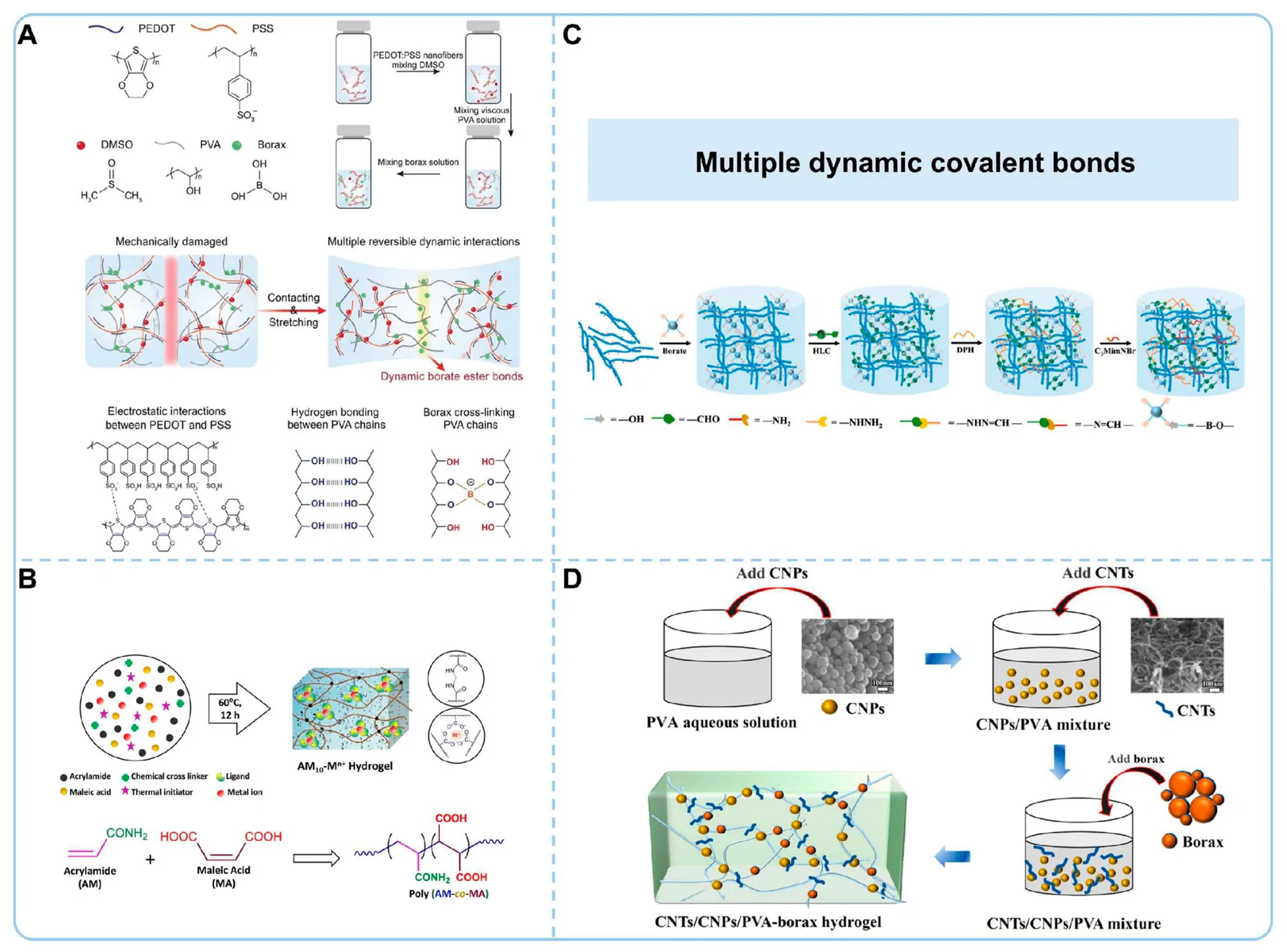
Self-healing mechanism classification of hydrogel sensors: (**A**) Synergistic effect of hydrogen bonds and boronate esters in the PEDOT:PSS-PVA nanocomposite network for restoring mechanical and electrical continuity after damage. Reproduced with permission from Ref. [120]. Copyright *Nanomaterials*. (**B**) Dynamic Ca^2+^–dicarboxylate ligand metal coordination in poly(acrylamide-co-maleic acid) network to achieve cartilage-like fracture energy. Reproduced with permission from Ref. [123]. Copyright *ACS Omega*. (**C**) Multiple dynamic covalent crosslinking in ionic liquid-based hydrogels integrating boronate ester, Schiff base and acylhydrazone chemistries. Reproduced with permission from Ref. [126]. Copyright *Gels*. (**D**) Nanocomposite-assisted self-healing mediated by CNT/CNP filler interfacial reconnection in PVA–borax matrix, enabling priority recovery of electrical properties. Reproduced with permission from Ref. [129]. Copyright *Gels*.

The stability challenges discussed in this section are directly connected to the network design strategies introduced in Section 2.1. Water loss, swelling, drying, freezing, chemical degradation, and conductivity changes all depend on crosslinking chemistry, solvent composition, and filler-matrix interactions. The properties reviewed in the preceding sections—architecture, mechanism, and enhancement—determine what a hydrogel sensor achieves during its first hours of operation. The properties reviewed in this section determine whether it is still working months later. Two degradation channels dominate field failures: environmental attack, in which freezing, dehydration, and swelling physically destroy the water-rich network; and mechanical damage, in which cuts, tears, and fatigue fractures sever conductive pathways and structural continuity. The corresponding defenses—anti-freezing and anti-dehydration formulation on the one hand, self-healing chemistry on the other—were historically treated as separate research directions, but the field has progressively recognized them as two faces of a single design problem: sustaining a functioning ion-transport network under conditions that thermodynamically favor its destruction. This section reviews the principal strategies for each defense (Figure 8 and Figure 9) and closes by examining how contemporary systems couple them.

### 5.1. Environmental Stability via Mixed Solvents and Organogels

The physics of freezing and drying damage. Water is both the essence and the vulnerability of the hydrogel platform. As the temperature approaches 0 °C, supercooled water nucleates ice crystals that grow by rejecting solutes, piercing the polymer network with expanding crystals, and locally desiccating the matrix; upon thawing, the melted regions leave voids and collapsed domains, so that a single freeze–thaw excursion can permanently degrade both mechanics and conductivity. Dehydration attacks the network more insidiously: evaporative water loss concentrates ions, stiffens the matrix, and shrinks the volume until the sensor detaches from skin and the conductive paths consolidate into resistive aggregates. A wearable sensor deployed across winter–summer transitions or stored in packaging experiences both. The anti-freezing literature accordingly pursues three formulation-level strategies, each manipulating the same thermodynamic variables—freezing point, water activity, and ion mobility—by different chemical means.

Solvent replacement strategies. The oldest and most direct strategy replaces a fraction of network water with an organic cosolvent that hydrogen-bonds with water more strongly than water bonds to itself, depressing the freezing point colligatively and disrupting ice nucleation kinetics. Ethylene glycol, glycerol, DMSO, and their mixtures are the workhorses, and binary water/cosolvent ratios between 30 and 60 volume percent typically extend frost tolerance to −20 to −60 °C. The design frontier lies in retaining conductivity, adhesion, and skin compliance alongside the solvent swap, because most cosolvents plasticize the network and raise ionic resistance. Conductive hydrogels designed with synergistic hydrophobic frameworks and dual antifreeze strategies illustrate the contemporary solution (Figure 8A) [107]: a stearyl methacrylate/CTAB hydrophobic association framework supplies mechanical integrity that survives cosolvent plasticization, while pyrrole/polyacrylic acid/ethylene glycol incorporation performs the dual duty of antifreezing and conductivity—the dual-antifreeze designation reflecting the combined action of glycol freezing-point depression and hydrophobic-domain water confinement. The resulting gels remain flexible, adhesive, and responsive at subzero temperatures while serving as wearable sensors through seasonal temperature excursions [108,109,110]. The residual limitations are familiar: cosolvent volatility is lower than water’s but nonzero, so long-term dehydration is delayed rather than eliminated, and the biological compatibility of high glycol fractions requires case-by-case verification for extended skin contact.

Salt incorporation strategies. Dissolved salts depress the freezing point colligatively while simultaneously supplying the mobile ions that the sensor requires for conduction—an elegant dual functionality that has made salt-loading the most widely adopted anti-freezing route. The mechanistic subtlety is ionic specificity: Li^+^, with its small radius and dense hydration shell, binds water so strongly that its solutions remain liquid to −70 °C and below, whereas the same molality of Na^+^ or K^+^ freezes at substantially higher temperatures. Low-temperature one-pot fabrication of dual-ion conductive hydrogels exploits precisely this chemistry (Figure 8B) [111]: Ca^2+^/Li^+^ salts ionically crosslink an acrylamide/gelatin network during a single gelation step performed at −20 °C, so that the gel forms at a temperature where conventional systems are already frozen solid. The dual-ion formulation assigns complementary roles—Ca^2+^ bridges carboxylate groups on gelatin chains into load-bearing junctions, while Li^+^ remains mobile and maintains ionic transport—and the resulting sensors operate directly on ice surfaces, monitoring the activity of humans and wild animals in winter field conditions [111]. High-concentration saline systems push the same physics to its limit: molten-salt-hydrate cellulose gels tolerate −80 °C while retaining measurable conductivity, at the cost of corrosivity that constrains electrode and packaging choices [112,113,114]. The trade-off structure of salt-loading is therefore well characterized: maximal frost tolerance and conductivity arrive together, but the deliquescent tendency of concentrated salts absorbs atmospheric moisture and causes dimensional drift in humid climates—the mirror image of the dehydration problem the cosolvent strategy addresses.

Deep eutectic solvents (DESs) and ionic liquids. The third strategy abandons the colligative logic of additives and reconstitutes the solvent phase itself. Deep eutectic solvents (DESs)—eutectic mixtures of hydrogen-bond donors and acceptors such as choline chloride/urea or choline chloride/imidazole—freeze tens of degrees below the melting points of either pure component, while their negligible vapor pressure eliminates evaporative loss entirely. Highly conductive, anti-freezing hemicellulose-based hydrogels prepared via choline chloride/imidazole DES dissolution of hemicellulose followed by ultraviolet polymerization of acrylamide demonstrate the full sustainability advantage of this route (Figure 8C) [115]: the hemicellulose feedstock is a lignocellulosic biomass side-stream, the DES simultaneously dissolves and plasticizes it, and the resulting gels combine cryogenic operation with water-insensitivity because the solvent phase contains essentially no free water to freeze or evaporate. Ionic-liquid gels generalize the same advantages with even wider electrochemical windows, and DES/ionic-liquid systems now hold the records for combined freeze–drought tolerance, sustaining operation from −80 to +80 °C with conductivity retention over months [116,117,118,119]. Their limitations are the mirror of their strengths: the hydrophobic or amphiphilic solvent phase weakens the hydrogen-bond network that gives PVA- and polysaccharide-based gels their toughness, so DES systems typically require explicit dynamic-covalent or coordination crosslinking to recover mechanical performance, and the biological profile of imidazolium-family ionic liquids demands careful toxicological screening for skin-facing applications.

Comparative assessment. The three strategies are complementary rather than competitive, and the choice follows the deployment envelope. Cosolvent systems suit moderate frost tolerance (−20 to −40 °C) with maximal skin compatibility and cost economy. Salt-loaded systems maximize frost tolerance and conductivity simultaneously and suit short-duration high-performance applications where dimensional drift is tolerable. DES and ionic-liquid systems are the only single-phase answer to combined freeze–drought–time exposure, and their emerging dominance in long-lifetime demonstrations reflects this completeness. The most capable contemporary formulations combine elements of all three—a glycol-plasticized, salt-loaded, partially DES-exchanged network—though such quadruple systems push formulation complexity toward the limits of manufacturable reproducibility.

### 5.2. Chemical and Physical Self-Healing Mechanisms

Why self-healing, and how it is measured. Self-healing chemistry has been introduced in Section 2.1; this section focuses on healing mechanisms and performance metrics. Wearable sensors accumulate damage through channels that no toughness enhancement can fully prevent: sharp-object cuts during daily wear, delamination at electrode interfaces, fretting fatigue at strain-concentrating features, and the slow coarsening of networks under sustained load. Self-healing chemistry addresses these events by endowing the network with reversible bonds that spontaneously reform across a damaged interface, restoring both mechanical continuity and, critically for sensing, electrical continuity. The literature evaluates healing along two axes whose decoupling matters: mechanical healing efficiency—the ratio of healed to pristine fracture strength or toughness—and electrical healing efficiency, the ratio of healed to pristine conductivity. Networks of high bond mobility heal electrically within seconds (ions reconnect across the interface almost immediately) but heal mechanically over minutes to hours, and the timescale mismatch defines the practical recovery protocol: a sensor whose leads are cut mid-measurement may resume signal transmission instantly while its load-bearing recovery completes overnight. Four healing chemistries dominate the current literature, differing in bond strength, exchange kinetics, and environmental tolerance.

Hydrogen-bond and borate ester synergism. The weakest and fastest healing chemistry relies on dense multiplexed hydrogen bonding, typically reinforced by dynamic borate ester bonds between borax and the vicinal diols of PVA. Self-healable PEDOT:PSS–PVA nanocomposite hydrogels exemplify the formulation logic (Figure 9A) [120]: DMSO-secondary-doped PEDOT:PSS nanofibers supply electronic conduction, viscous PVA chains supply the diol substrate for borax crosslinking, and the interplay of PVA hydrogen bonds, PEDOT:PSS electrostatic complexes, and dynamic borate ester bonds creates a network in which multiple weak interactions collectively restore both mechanical integrity and electrical pathways within minutes of cutting. The multiplicity of interactions is the design essence: any single hydrogen bond is too weak and too short-lived to bridge an interface, but thousands acting in parallel produce macroscopic healing with effectively instantaneous kinetics. The corresponding limitation is thermal: hydrogen-bond and borate networks soften and creep at physiological-to-elevated temperatures, and their healing efficiency degrades as healing repeats, because each cut severs some fraction of chains permanently [121,122]. They suit applications where the dominant damage mode is superficial cutting rather than structural tearing.

Dynamic metal–ligand coordination. Coordination bonds between multivalent metal ions and chelating ligands occupy the middle ground of the strength–kinetics spectrum: stronger than hydrogen bonds, they heal more slowly but recover more toughness. The canonical AM_10_-M^n+^ hydrogels—formed by one-pot copolymerization of acrylamide and maleic acid followed by Ca^2+^ coordination of the dicarboxylate ligands—exhibit cartilage-like fracture energies near 1500 J m^−2^, 15–16-fold stretchability, and fast self-healing, alongside strain sensing, supercapacitor, and electromagnetic-shielding multifunctionality (Figure 9B) [123]. The mechanistic richness of coordination healing lies in its tunability: ligand field strength, metal identity (Ca^2+^, Zn^2+^, Fe^3+^, Al^3+^), and coordination stoichiometry jointly set the bond lifetime, and the exchange kinetics that govern healing rate can be tuned over orders of magnitude without touching the network topology. Ferric-triphosphate complexes push the strength ceiling while zinc-carboxylate pairs optimize kinetics; antifreezing variants incorporate CaCl_2_ into the same coordination network, unifying the self-healing and environmental-adaptation agendas in a single formulation [124,125]. The principal weakness is competitive displacement: biological fluids and sweat contain their own chelators—citrate, phosphate, proteins—that can strip the metal from the network over time, a degradation channel unique to this chemistry class.

Multiple dynamic covalent bonds. The strongest healing chemistry deploys reversible covalent linkages—borate esters, Schiff bases, acylhydrazones, Diels–Alder adducts—whose bond energies approach permanent covalency while retaining exchange capability under specific triggers. Ionic-liquid-based hydrogels crosslinked simultaneously by borate ester, Schiff base, and acylhydrazone bonds demonstrate the current state of the art (Figure 9C) [126]: the three orthogonal chemistries activate at different points of the deformation and healing cycle—borate esters exchange fastest, Schiff bases provide intermediate-strength reversible junctions, and acylhydrazones carry the highest load—and their cooperation yields stretchable, rapidly self-healing, antibacterial gels with healing efficiencies exceeding 90% within minutes. The orthogonality is the design lesson: rather than seeking a single ideal bond, the network distributes its reversible junctions across the accessible energy spectrum so that healing proceeds on multiple timescales simultaneously, fast bonds re-bridging the interface while slow bonds restore strength. The costs are synthetic complexity and trigger dependence—acylhydrazone exchange is acid-catalyzed, Diels–Alder reversion is thermal—so the healing conditions must be engineered as deliberately as the bonds themselves [127,128].

Nanocomposite-assisted healing. The fourth strategy integrates conductive nanofillers not merely as passive reinforcement but as active participants in the healing process. CNTs/CNPs/PVA–borax conductive self-healing hydrogels disperse carbon nanotubes and carbon nanoparticles within a borax-crosslinked PVA matrix (Figure 9D) [129]: the borate ester equilibrium provides the reversible crosslinking, while the carbon fillers—surface-functionalized with oxygen groups that hydrogen-bond to PVA—serve as multivalent junction mediators whose surfaces nucleate interface re-bridging after damage. The electrical consequences are distinctive: because the filler network reconnects as soon as the two fracture surfaces contact, electrical healing precedes mechanical healing by minutes, and the sensor resumes signal transmission while its load-bearing recovery is still in progress. The filler surface chemistry, hydrogen-bonding density, and percolation architecture jointly govern the healing kinetics and the recovered conductivity level, with higher filler loadings accelerating electrical recovery at some cost to healing completeness, since rigid filler-rich zones resist re-coalescence [130,131]. Nanocomposite healing thus offers a performance profile—electrical-first recovery with tunable mechanical follow-through—uniquely suited to sensing applications where signal continuity matters more than structural restoration.

Comparative assessment. The four chemistries sort cleanly along the strength–speed plane. Hydrogen-bond networks heal fastest and weakest, suited to high-frequency superficial damage. Coordination networks offer the best balance for most wearable scenarios and uniquely unify with anti-freezing salt chemistry. Multiple-dynamic-covalent systems deliver the highest recovered strength for structural applications, at synthetic cost. Nanocomposite systems prioritize electrical recovery and compose naturally with any of the other three, which is why the most cited recent designs pair a carbon-filler network with a coordination or dynamic-covalent matrix. Across all four, the open problem is healing under load: virtually all reported systems heal quiescently, with the fracture surfaces held in contact and unstressed, whereas field damage occurs while the device is worn, stretched, and moving. Demonstrating healing-under-service remains the discipline’s most conspicuous gap between laboratory and deployment. A consistent comparison of healing time, healing conditions, mechanical healing efficiency, electrical healing efficiency, number of healing cycles, and mechanical and electrical recovery is provided in Table 5.

### 5.3. Coupled Design for Long-Term Robustness

The anti-freezing and self-healing agendas converge in a design philosophy that views the hydrogel sensor as a chemical system whose components must collectively buffer every degradation channel simultaneously: freezing, drying, cutting, fatiguing, and fouling. The convergence is visible in the literature’s most capable recent systems—salt-loaded coordination networks that heal and resist frost in one formulation; DES-plasticized dynamic-covalent gels that neither freeze nor dry nor lose conductivity across months; nanocomposite ionogels whose fillers restore electrical continuity while the DES phase insulates the network from ambient water exchange. Two design principles generalize these successes. First, multifunctional components beat additive ones: the best formulations assign each chemical species several simultaneous roles (Ca^2+^ as both crosslinker and antifreeze; borate as both healer and diol-binder; MXene as both conductor and water-retainer), because additive stacking of single-function components accumulates incompatibilities. Second, degradation channels interact, and defenses must be co-designed rather than concatenated: a salt-loaded gel that heals in humid summer conditions may leach and stiffen in dry winter ones; a DES gel immune to water loss may suffer interfacial fouling that blocks healing contact. The next generation of robust hydrogel sensors will accordingly be designed less as gels-with-additives and more as integrated chemical economies in which every species earns its place across multiple stress scenarios.

## 6. Applications of Gel Materials in Wearable Electronics and Robotics

The material architectures, transduction mechanisms, and robustness strategies reviewed in Section 2, Section 3, Section 4 and Section 5 acquire technological meaning only through application. This section surveys the deployment landscape of hydrogel sensors along three application frontiers with progressively larger systems scope: health monitoring biosensing, where the sensor interrogates the chemistry of the body; electronic skin and human–machine interaction, where the sensor mediates between human intention and machine response; and emerging scenarios—rehabilitation, underwater operation, and AI-fused interaction—that stress multiple sensor capabilities simultaneously. A consistent theme across all three frontiers is the migration from single-sensor demonstrations toward integrated systems: sensing arrays paired with signal conditioning, wireless telemetry, and increasingly with machine-learning inference, so that the hydrogel element, however sophisticated its chemistry, is one link in a measurement chain whose end-to-end performance is what applications actually demand. To identify which hydrogel systems currently show the greatest potential for practical wearable applications, we compare representative material families across the criteria that matter most for real deployment: stretchability, conductivity, self-healing ability, environmental stability, biocompatibility, manufacturability, and integration with readout electronics. Table 6 summarizes this comparison. Table 7 summarizes representative hydrogel sensors and their key performance metrics.

Double-network hydrogels offer high toughness and fatigue resistance, but their multi-step synthesis and limited conductivity can complicate scale-up. Semi-interpenetrating networks provide a useful decoupling of conductivity and mechanics, but long-term stability under sweat and humidity remains a concern. Organohydrogels and mixed-solvent gels show strong potential for wide-temperature operation because they resist freezing and drying, but their conductivity and skin compatibility depend strongly on solvent choice. MXene composite hydrogels combine high conductivity with mechanical reinforcement, but oxidative degradation and batch-to-batch variability remain obstacles. Polyrotaxane hydrogels exhibit outstanding stretchability, softness, and cyclic stability, but their synthesis is complex and cost remains high. Ionogels and deep eutectic solvent gels offer excellent environmental stability, yet their biocompatibility and mechanical toughness require further optimization. Carbon-based composite hydrogels benefit from mature chemistry and low cost, but their sensitivity is generally lower than that of MXene composites. Liquid metal composite hydrogels provide ultrahigh stretchability and self-healing, but their long-term biodistribution and encapsulation requirements remain unresolved.

On balance, organohydrogels, MXene composites, and polyrotaxane hydrogels currently show the greatest potential for practical wearable applications, provided that long-term stability, biocompatibility, and manufacturing reproducibility are addressed. For near-term commercialization, organohydrogels and semi-interpenetrating networks are the most promising because their processing is relatively mature and their performance can be tuned through formulation. For high-performance applications such as electronic skin and human–machine interaction, MXene composites and polyrotaxane hydrogels offer the most attractive combination of sensitivity, stretchability, and cyclic stability. Table 6 summarizes this comparison.

**Table 6:** Comparison of representative hydrogel systems for practical wearable applications.

Material System	Stretchability and Conductivity	Self-Healing and Environmental Stability	Biocompatibility, Manufacturability, and Practical Wearable Potential	References
Double-network hydrogels	High stretchability; moderate conductivity	Limited self-healing; moderate environmental stability	Good biocompatibility and moderate manufacturability; high toughness and suitable for strain sensing, but scale-up challenging	[11,23,24,25]
Semi-interpenetrating networks	Moderate to high stretchability; moderate to high conductivity	Limited self-healing; moderate environmental stability	Good biocompatibility and good manufacturability; good balance of conductivity and mechanics, but humidity stability needs improvement	[26]
Organohydrogels and mixed-solvent gels	High stretchability; moderate conductivity	Moderate self-healing; excellent environmental stability	Component-dependent biocompatibility and good manufacturability; strong potential for wide-temperature wearable sensors	[21,27]
MXene composite hydrogels	High stretchability; high conductivity	Good self-healing; moderate environmental stability	Dose-dependent biocompatibility and moderate manufacturability; high sensitivity and conductivity, but oxidation and batch variability remain issues	[22,37,38]
Polyrotaxane hydrogels	Very high stretchability; moderate conductivity	Limited self-healing; good environmental stability	Good biocompatibility but low manufacturability; excellent stretchability and cyclic stability, but complex synthesis	[12]
Ionogels and deep eutectic solvent gels	High stretchability; high conductivity	Good self-healing; excellent environmental stability	Requires biocompatibility screening and moderate manufacturability; excellent environmental stability, but biocompatibility and toughness need optimization	[114,115,116,117,118]
Carbon-based composite hydrogels	Moderate to high stretchability; high conductivity	Moderate self-healing; moderate environmental stability	Good biocompatibility and good manufacturability; mature materials, but sensitivity lower than MXene composites	[13,14,15,128]
Liquid metal composite hydrogels	Very high stretchability; high conductivity	Excellent self-healing; good environmental stability	Requires encapsulation for biocompatibility and moderate manufacturability; ultrahigh stretchability and self-healing, but long-term biodistribution unclear	[132]

**Table 7:** Summary of representative hydrogel sensors and key performance metrics.

Material System	Sensing Mechanism and Sensitivity	Detection Range and Response/Recovery Time	Stability, Self-Healing, and Application	References
PAA/LCNF/SA double network	Piezoresistive; gauge factor not uniformly reported	Strain sensing; response time not uniformly reported	Moderate stability; limited self-healing; strain sensor	[23]
PVA/ANF/NaCl organohydrogel	Piezoresistive; high cyclic stability	Distance, pressure, strain; response time not uniformly reported	Excellent environmental stability; limited self-healing; wearable sensor and handwriting recognition	[27]
MXene/PVA/PAM	Piezoresistive; high sensitivity	2800% strain; 90 ms response and 240 ms recovery	Moderate stability; good self-healing; electronic skin	[22]
MXene/MMT composite	Piezoresistive; high sensitivity	Wearable strain; response time not uniformly reported	Moderate stability; limited self-healing; wearable sensor and supercapacitor	[37]
Layered PVA/MXene	Piezoresistive; tensile strength 2.87 MPa	Underwater sensing; response time not uniformly reported	Good stability; limited self-healing; underwater sensing	[38]
PEDOT:PSS-PVA	Piezoresistive; gauge factor not uniformly reported	Human motion; healing within minutes	Moderate stability; good self-healing; human motion monitoring	[119]
Polyrotaxane hydrogel	Piezoresistive; 2450% stretchability	Motion and electrophysiology; 10,000 cycles	Good stability; limited self-healing; wearable motion and ECG/EMG	[12]
SA/PAM anti-swelling	Piezoresistive; conductivity 1.2–1.6 S m^−1^	Underwater motion; 72 h immersion	Good stability; limited self-healing; underwater communication	[133]
Ionic thermoelectric hydrogel	Thermoelectric; thermopower 24.17 mV K^−1^	Temperature gradient; response time in seconds	Moderate stability; limited self-healing; thermal mapping	[67]
Triboelectric ionic hydrogel	Triboelectric; open-circuit voltage	Tactile; response time in milliseconds	Moderate stability; limited self-healing; tactile sensing	[66]
Electrochemical aptamer microneedle	Electrochemical; detection at µM–nM	Drug monitoring; response time in minutes	Moderate stability; limited self-healing; biosensing	[68]
Multimodal stacked hydrogel	Multimodal; decoupling accuracy	Multi-stimulus; response depends on stack	Moderate stability; limited self-healing; multimodal sensing	[69]

### 6.1. Functional Gels for Health Monitoring Biosensing

**Figure 10:**
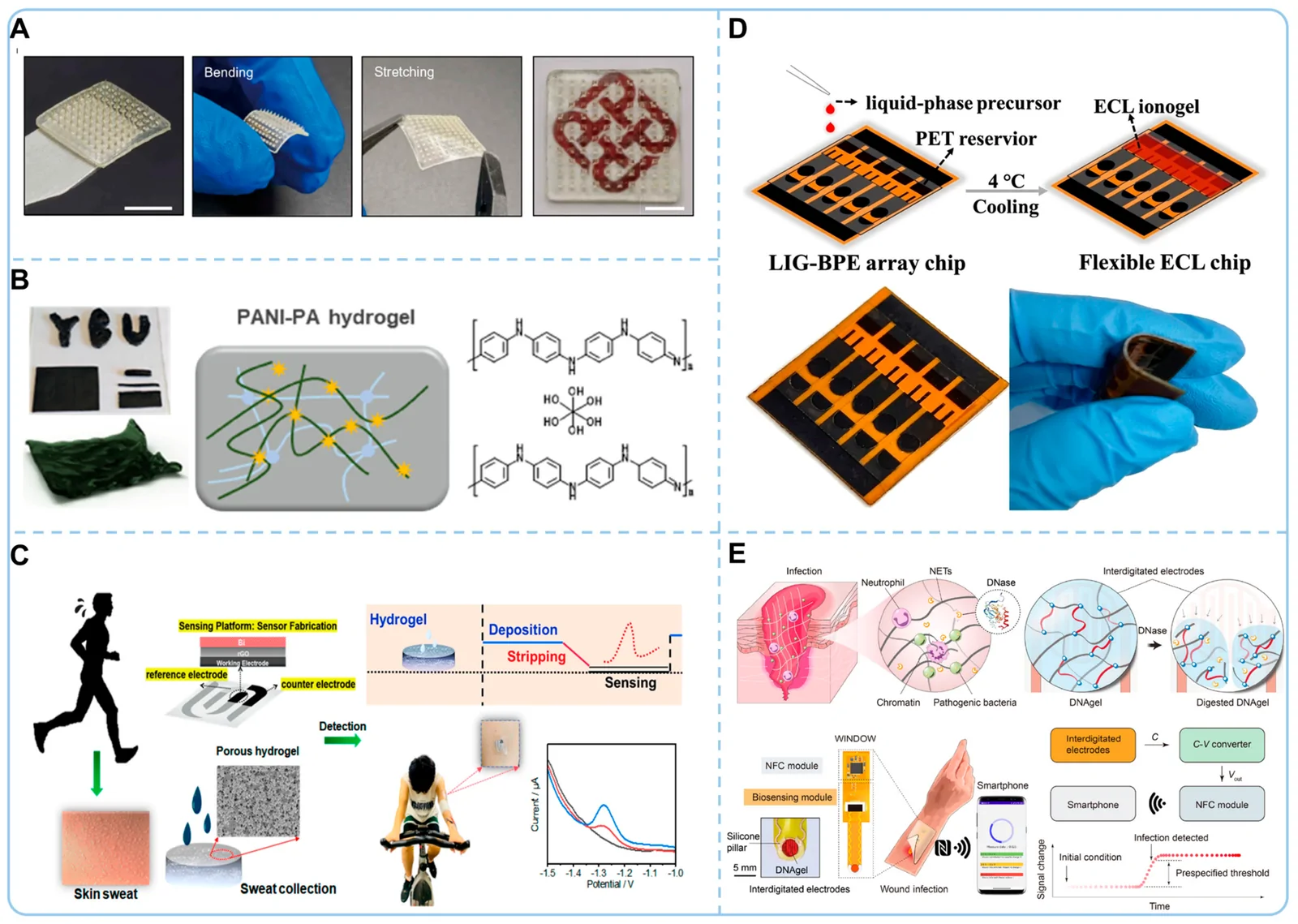
Wearable biosensing patch architecture across the health-monitoring hierarchy: (**A**) Ultrasound-readable microneedles for continuous glucose monitoring based on covalently labeled ultrasound contrast agents. Reproduced with permission from Ref. [134]. Copyright *Science Advances*. (**B**) Hydrogel-based flexible pH fiber sensor relying on protonation-dependent conductivity of polyaniline for real-time sweat pH tracking. Reproduced with permission from Ref. [136]. Copyright *Gels*. (**C**) Wearable patch enhanced by magnetic porous hydrogel for sweat zinc ion quantification via anodic stripping voltammetry. Reproduced with permission from Ref. [137]. Copyright *Sensors*. (**D**) Fully flexible electrochemiluminescence chip integrating laser-induced graphene dual electrodes, deep eutectic solvent ionogel and microfluidic sweat collector for smartphone-readout metabolite detection. Reproduced with permission from Ref. [138]. Copyright *Advanced Science*. (**E**) Wireless and battery-free wound infection sensor based on DNA hydrogel combined with interdigitated electrodes and near-field communication smartphone readout. Reproduced with permission from Ref. [141]. Copyright *Science Advances*.

Continuous glucose monitoring augmented by ultrasound. Diabetes management remains the canonical application of wearable biochemical sensing, and hydrogel microneedle technology has progressively displaced fingerstick sampling for interstitial glucose. A representative example illustrates how a sensing chemistry and a readout modality can be co-designed to eliminate the most constraining element of conventional continuous glucose monitors—the wired electrochemical readout. A microneedle patch covalently labeled with ultrasound contrast agents reports interstitial glucose acoustically: the contrast agents bind glucose competitively, and their acoustic backscatter shifts in a glucose-dependent manner, so that a wearable ultrasound probe worn over the patch reconstructs glucose kinetics without any electrical connection to the sensing site (Figure 10A) [134]. The design philosophy generalizes: by converting the chemical signal into a physical observable that consumer devices already measure—acoustic, optical, magnetic—the biosensor sheds its custom electronics and rides on existing hardware infrastructure. Complementary continuous-monitoring strategies based on glucose-oxidase microneedles, optical boronate chemistries, and closed-loop insulin delivery continue to mature along the electrochemical path [135].

Sweat analysis: the richest non-invasive modality. Sweat offers what blood and interstitial fluid cannot: truly non-invasive, continuously renewable access to a biofluid carrying electrolytes, metabolites, hormones, and heavy metals. Three systems illustrate the maturing hierarchy of sweat sensing capability. At the single-analyte level, PANI-PA-PVA hydrogel fiber sensors monitor sweat pH in real time through the protonation-state-dependent conductivity of polyaniline; the fiber format allows direct weaving into headbands and wristbands where sweat first accumulates (Figure 10B) [136]. At the ion-quantification level, magnetic porous hydrogel patches combine magnetic-assisted sample routing—magnetite nanoparticles in the hydrogel matrix actively wick sweat toward the electrode—with anodic stripping voltammetry for trace zinc quantification, a design that solves the sample-delivery bottleneck electrochemical sensing faces in the low-sweat-rate regime (Figure 10C) [137]. At the systems level, all-soft electrochemiluminescence (ECL) chips integrate laser-induced graphene bipolar electrode arrays, deep eutectic solvent ionogels, and microfluidic sweat collectors into a disposable platform that quantifies multiple sweat metabolites at $0.106 per chip with smartphone-camera readout, replacing a benchtop photomultiplier-tube instrument with the camera nearly every user already carries (Figure 10D) [138]. The trajectory across the three systems is unmistakable: from specialized sensing chemistry, through sample-management engineering, toward complete measurement systems whose cost and usability profiles fit consumer deployment. Broader sweat-sensing platforms extend the analyte menu to lactate, cortisol, caffeine, and heavy metals, and osmotic-harvesting patches that extract sweat passively without exercise broaden the user population beyond athletes [139,140].

Wound infection detection. Wound monitoring closes the biosensing loop between physiological measurement and therapeutic decision. A wireless, battery-free sensor based on DNase-responsive DNA hydrogels detects bacterial infection directly at the wound site: pathogenic bacteria secrete DNases that degrade the DNA hydrogel crosslinks, collapsing the gel and shifting the impedance of an interdigitated electrode pair; a near-field-communication coil relays the impedance state to a smartphone, which triggers an alert when the wound crosses the infection threshold (Figure 10E) [141]. The design deserves note for what it omits: no battery, no onboard microprocessor, no analog front end—just a stimulus-responsive hydrogel, an electrode, and an NFC antenna. It is the clearest demonstration that hydrogel chemistry, when engineered as a transducer, can absorb the functions that conventional sensing assigns to electronics, and it prefigures a family of “material-as-instrument” medical sensors whose unit cost approaches that of a bandage.

Assessment of the biosensing frontier. Across glucose, sweat, and wound applications, three system-level requirements now dominate over chemistry-level innovation: calibration stability over weeks of continuous wear, sample access that does not depend on user behavior, and regulatory-grade validation of accuracy against reference methods. The most capable systems solve these jointly—the ultrasound-readable patch by removing drift-prone electrochemistry, the ECL chip by making each measurement a fresh single-use device, the wound sensor by designing the chemistry to survive the wound environment itself. The next wave of biosensing systems will be differentiated less by novel recognition chemistries and more by robustness engineering of the complete measurement chain.

### 6.2. Gel-Based Electronic Skin and Human–Machine Interaction

**Figure 11:**
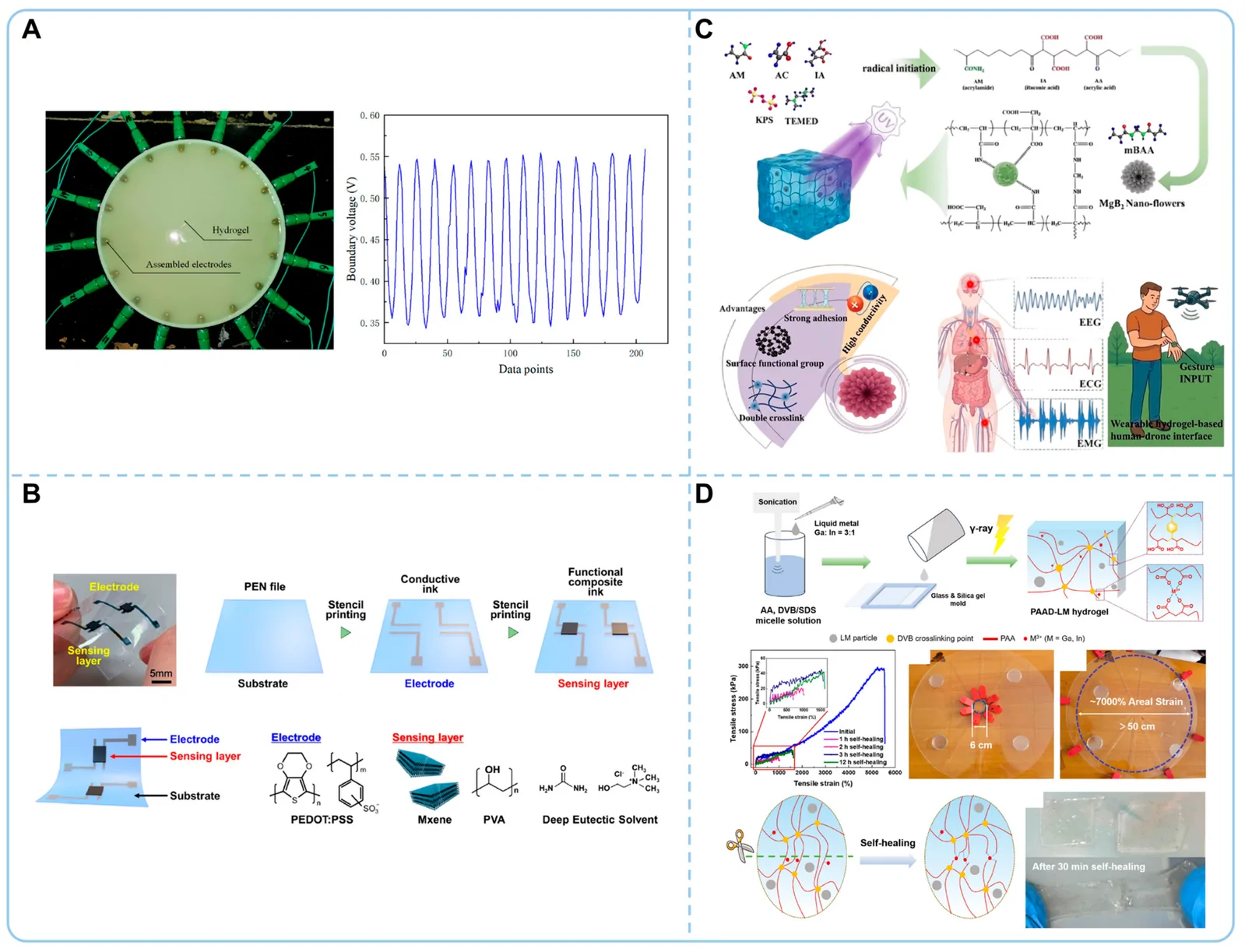
Electronic skin and human–machine interaction devices: (**A**) Multi-point tactile-sensing electronic skin based on electrical impedance tomography, reconstructed using hydrogel materials and the FISTA algorithm. Reproduced with permission from Ref. [142]. Copyright *Sensors*. (**B**) MXene-based high-performance soft pressure sensor adopting gel–deep eutectic solvent composite and screen-printed electrodes for tactile mapping. Reproduced with permission from Ref. [143]. Copyright *Micromachines*. (**C**) Hierarchically synergistic ionoelectronic hydrogel combined with convolutional neural network for flexible sensing and UAV gesture control with an accuracy of 92.15%. Reproduced with permission from Ref. [144]. Copyright *Nano Research*. (**D**) Super-stretchable hydrophobic/electrostatic dual-crosslinked poly(acrylic acid)–liquid metal hydrogel touch panel with areal strain >7000%, for self-healable tactile interaction and virtual reality gloves. Reproduced with permission from Ref. [145]. Copyright *npj Flexible Electronics*.

Tactile perception: from single pixels to tomographic maps. Electronic skin replicates the distributed tactile perception of human skin, and its central engineering tension is between spatial resolution and wiring density: a conventional electrode grid reads each taxel independently, so resolution scales linearly with wire count, and high-resolution skins drown in cabling. Electrical impedance tomography (EIT) inverts this scaling by reconstructing the interior conductivity distribution of the sensing skin from boundary measurements alone (Figure 11A) [142]: hydrogel electrodes around the skin’s perimeter inject currents and measure boundary voltages, and a FISTA-algorithm reconstruction maps the conductivity field—so that the number of wires stays fixed at the perimeter while spatial resolution is limited only by the reconstruction inverse problem. The hydrogel contributes essential properties to this scheme: its conductivity changes strongly and locally under pressure, and its mechanical compliance keeps boundary electrodes in stable contact through deformation. Complementing the tomographic approach, MXene-based soft pressure sensors employing gel–deep eutectic solvent composites pair stencil-printed electrodes with MXene/PVA/DES sensing layers, achieving high-performance tactile mapping in the conventional array format where maximum per-taxel fidelity is the priority (Figure 11B) [143]. The two architectures mark the design space’s poles—tomographic minimalism versus array maximalism—with hybrid hierarchical layouts increasingly borrowing from both. Related tactile technologies span resistive arrays, piezoelectric films for dynamic touch, and proximity-sensing ionic skins that detect approaching objects before contact [20].

Gesture control and machine-learning interaction. Gesture recognition converts distributed strain signals into machine commands, and its recent progress has come less from sensor chemistry than from the fusion of sensor arrays with convolutional neural networks. The coordinated hierarchical ion–electron hydrogel system demonstrates the full pipeline (Figure 11C) [144]: a magnesium-boride-nanostructure-reinforced hydrogel worn on the hand streams strain signals from finger flexion; a CNN trained on the signal patterns classifies gestures at 92.15% accuracy; and the classified gesture drives drone posture control in real time. The architectural novelty lies in the coordinated ion–electron conduction—the hydrogel maintains ionic conduction for skin compliance while the nanostructures supply an electronic pathway for low-noise readout, and the CNN tolerates the residual noise that purely ionic systems exhibit. Parallel developments extend the paradigm across modalities: deep-learning pipelines translate hydrogel array signals into sign-language sentences, air-writing characters, and rehabilitation-assessment scores, in each case exploiting the network’s ability to learn the crosstalk that Section 3.6 identified as the fundamental obstacle to single-signal interpretation [132]. The pattern is a harbinger for the field: machine learning is becoming the standard answer to the nonidealities of soft-matter sensing.

Stretchable touch panels: the interface endpoint. Where gestures suffice for commands, touch panels support the full vocabulary of graphical interaction—writing, drawing, pointing, clicking—and hydrogel implementations face the demanding combination of large-area stretchability, transparency, and multi-touch localization. The ultrastretchable, multifunctional hydrophobic/electrostatic dual-crosslinked PAAD-LM hydrogel represents a current benchmark (Figure 11D) [145]: synthesized by one-step γ-radiation copolymerization of acrylic acid, divinylbenzene, and liquid metal, the gel sustains over 7000% areal strain while functioning as a self-healing touch panel for painting, keyboard, and mouse control, and as an interface layer in virtual-reality smart gloves. The liquid metal domains confer two complementary properties—deformable electronic conduction that survives astronomical strains, and damage tolerance that heals electrical continuity after puncture—while the hydrophobic/electrostatic dual crosslinking protects the network against the swelling and fouling that defeat hydrophilic gels in daily handling. Related stretchable touch panels, on-skin touchless sensors, and proximity–pressure bimodal interfaces extend the interaction vocabulary toward the gesture-free, contactless interfaces that augmented reality demands [146,147].

Assessment of the HMI frontier. Electronic skin and human–machine interaction applications are converging on a common system template: a distributed hydrogel sensing layer, a flexible or wireless readout layer, and an inference layer whose machine-learning models absorb the nonideality of soft-matter signals. The differentiating questions have correspondingly shifted from materials to systems: How many sensing channels can a wearable interface sustain before wiring and power dominate? What recognition accuracy survives cross-user and cross-session variability? And how do interaction latencies stack up between sensing, inference, and actuation? The answers increasingly require co-design across all layers, which is why the strongest recent demonstrations—drone control at 92% accuracy, VR gloves surviving thousands of donning cycles—are systems papers with hydrogel chemistry at their core.

### 6.3. Bio-Inspired Actuators and Soft Robotics Based on Gels

Directional hydrogel crawling requires periodic swelling–contraction or bending–recovery cycles to be coupled with asymmetry. Body geometry can establish different front and rear contact forces, and directional friction interfaces can regulate sliding. Local stimulation can also alter contact between individual body regions and the substrate [94,95].

Liu and Zheng developed a caterpillar-inspired crawling robot based on a thermoresponsive bilayer hydrogel. A PNIPAM-based hydrogel and poly(vinyl alcohol)/sodium alginate (PVA/SA) components formed an anisotropic bilayer with differential responses (Figure 6A). Fe_3_O_4_ nanoparticles enhanced photothermal conversion and heat conduction [96]. Under illumination, unequal thermal contraction of the two layers bent the robot and established asymmetric substrate contact. Increasing curvature displaced the body forward. After illumination ceased, cooling and water reabsorption gradually restored the initial configuration, completing a crawling cycle. Adding Fe_3_O_4_ reduced the time required for 30° bending from approximately 1 h to 0.5 h. The robot achieved approximately 10 mm displacement per movement and a ground locomotion speed of approximately 54.5 mm h^−1^. This study demonstrates that differential thermal responses and interfacial design can convert the PNIPAM volume phase transition into directional ground locomotion. However, heat transfer and rehydration continued to limit motion. Recovery after illumination ceased still required approximately 6 min. Faster deswelling and reswelling therefore remain essential for increasing the locomotion frequency of thermoresponsive hydrogel robots.

Combining photothermal nanomaterials with digital manufacturing further improves localized actuation and motion programmability in hydrogel robots. Teng et al. incorporated photothermally active MoS_2_ into PNIPAM hydrogels and fabricated several bioinspired soft robots using digital light processing (DLP) three-dimensional printing [97]. A six-legged crawling robot used near-infrared light to induce reversible extension and contraction of the PNIPAM/MoS_2_ hydrogel. Differences in substrate contact and recovery between horizontal and prebent feet generated directional motion (Figure 6B). The original prebent feet produced little effective displacement because their length was poorly matched to material output force. Shortening the actuating feet and optimizing the structure allowed elastic restoring forces during recovery to propel the robot forward. Thus, hydrogel locomotion depends strongly on actuator dimensions and robot geometry, as well as on material response. The study also combined magnetic units with hydrogel structures to enable straight rolling, turning, and obstacle traversal under a rotating magnetic field. However, multiple stimuli and hybrid actuation also increase the complexity of material composition, external field equipment, and control strategies.

### 6.4. Emerging Scenarios: Towards Intelligent Gel Systems

**Figure 12:**
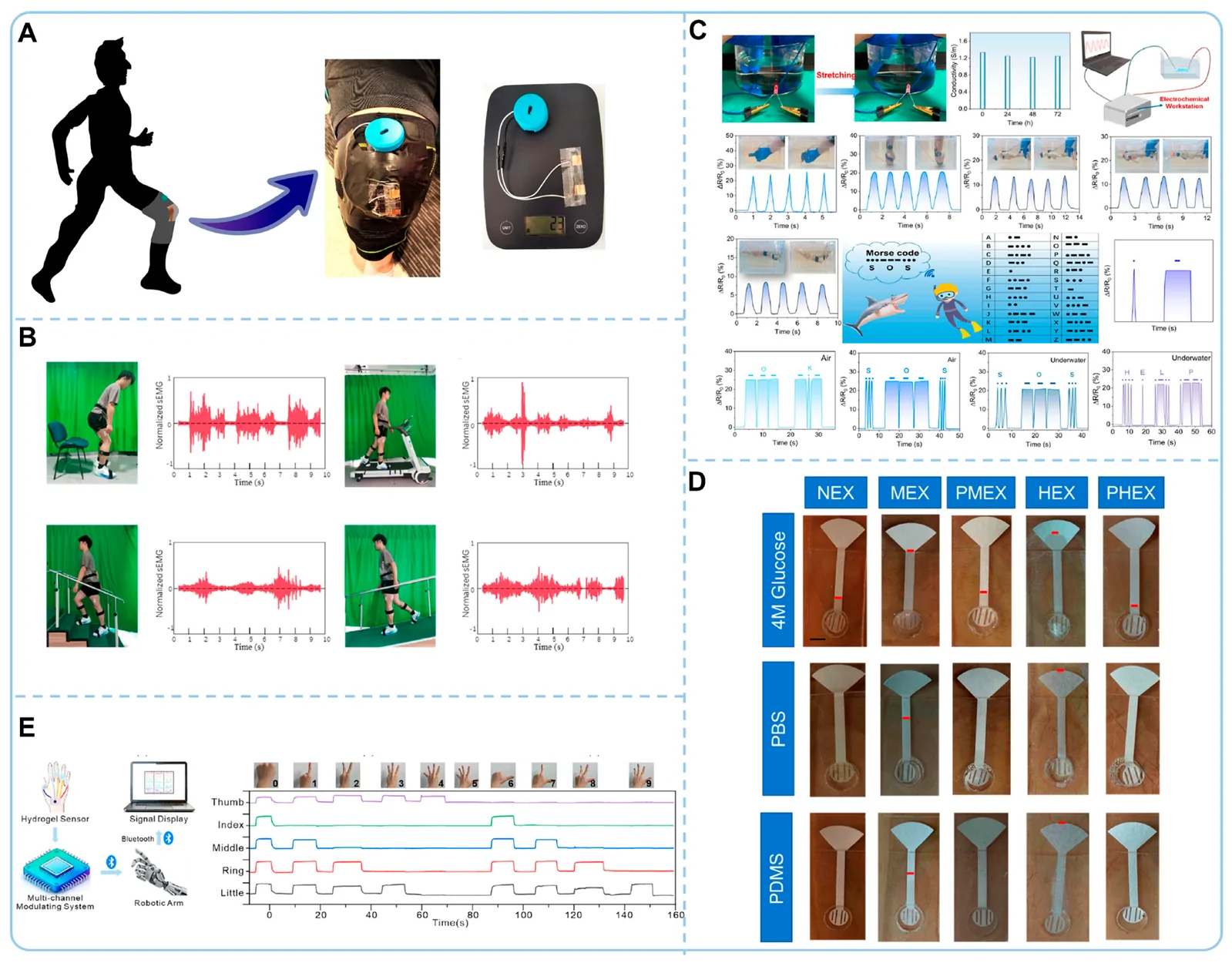
Application scenario panorama of hydrogel sensors: (**A**) AI-assisted wearable hydrogel sensing device for knee gait analysis, calibrated with an electronic scale for pathological gait discrimination. Reproduced with permission from Ref. [148]. Copyright *Sensors*. (**B**) Biointegrated conductive hydrogel for real-time surface electromyography motion sensing in exoskeleton-assisted lower-limb rehabilitation, covering standing, walking, stair climbing and downhill activities. Reproduced with permission from Ref. [133]. Copyright *Sensors*. (**C**) High-strength anti-swelling sodium alginate/polyacrylamide hydrogel strain sensor for underwater motion monitoring and Morse code information transmission, maintaining a conductivity of 1.2–1.6 S m^−1^ over 72 h. Reproduced with permission from Ref. [151]. Copyright *Gels*. (**D**) Osmosis-driven wearable patch for passive sweat sampling and lactate quantification. Reproduced with permission from Ref. [153]. Copyright *Micromachines*. (**E**) Stretchable mechanically interlocked polyrotaxane hydrogel for wearable motion and electrophysiological monitoring, with a five-sensor gesture recognition array enabling wireless robot control. Reproduced with permission from Ref. [12]. Copyright *Advanced Science*.

AI-assisted gait analysis. Gait is a diagnostic goldmine—Parkinson’s disease, hemiplegia, and fall risk all imprint on stride dynamics—but laboratory gait analysis requires camera motion-capture systems that confine assessment to clinics. Wearable hydrogel sensors relocate gait measurement to daily life, and the AI-aided gait analysis system demonstrates the complete pipeline (Figure 12A) [148]: a hydrogel strain sensor mounted at the knee captures the flexion-extension cycle of walking; calibration against an electronic scale establishes the force–response mapping; and machine-learning classification discriminates pathological gait patterns from healthy locomotion. The knee-mounted single-sensor approach is deliberately minimalist—against multi-sensor alternatives that instrument the whole limb—reflecting the clinical reality that sensor count trades directly against patient compliance. Motion-monitoring platforms of the same genre extend to gesture-recognition smart gloves and multimodal rehabilitation systems [149,150].

Exoskeleton-assisted rehabilitation. Lower-limb exoskeletons restore walking to patients with neurological impairment, but their assistance strategies need real-time knowledge of the patient’s residual effort to assist rather than replace it. Biointegrated conductive hydrogels supply that knowledge (Figure 12B) [133]: skin-mounted hydrogel electrodes acquire surface electromyography from the muscles driving each gait phase—standing up, constant-speed walking, stair climbing, downhill walking—with normalized sEMG traces resolving the distinct activation signatures of each activity, and the exoskeleton’s controller modulates torque assistance accordingly. The closed loop between biological effort and robotic assistance defines the emerging field of active rehabilitation, and hydrogel electrodes are its enabling interface technology because only they sustain the low-impedance, motion-artifact-tolerant skin contact that weeks-long rehabilitation protocols require. The broader rehabilitation-sensing agenda spans gesture-recognition training systems, multi-site sEMG arrays, and sensorized prosthetic liners that monitor residual-limb health.

Underwater operation. Underwater deployment stresses every vulnerability of hydrogels simultaneously—swelling dilutes ions, prolonged immersion softens networks, and conventional gels lose adhesion within hours—yet marine applications from diving communication to soft-robotic grasping demand exactly this capability. The high-strength, anti-swelling sodium alginate/polyacrylamide hydrogel demonstrates the formulation solution (Figure 12C) [151]: a dense dual network suppresses water ingress so effectively that conductivity holds between 1.2 and 1.6 S m^−1^ through 72 hours of immersion, and finger-, wrist-, elbow-, and knee-mounted sensors deliver stable cyclic resistance responses during underwater motion monitoring. The system’s most striking demonstration is communication: a diver wearing the sensors transmits Morse code by tapping—SOS and HELP sequences encoded in the dot–dash rhythm of resistance changes, distinguishable both in air and underwater—establishing a channel for situations where radio and acoustic links fail. Related underwater platforms include ionogel-based sensors and communicators whose nonaqueous solvent phase is intrinsically immune to swelling, and non-swelling MXene nanocomposite gels for subsea strain sensing [152].

Osmotic sweat harvesting and lactate quantification. Sweat-based biochemical sensing normally requires the user to sweat—exercise, heat, or iontophoresis—which restricts continuous monitoring to workouts and clinics. Osmotically enabled wearable patches eliminate the dependency (Figure 12D) [153]: a hydrogel core loaded with osmotic agents draws fluid from the skin itself through the osmotic pressure differential between the interstitial space and the patch, harvesting sweat at rest and delivering it to a lactate-oxidase electrode for continuous quantification. The osmotic strategy joins the family of sweat-access technologies—microfluidic capillaries, hydrophilic channel networks, magnetic routing—each solving the same problem of getting biofluid to the sensor without user behavior; its distinguishing virtue is passivity, since osmotic extraction requires no external energy or stimulus. Together with the analyte-detection systems of Section 6.1, osmotic harvesting completes a sweat-sensing stack that operates during ordinary daily life [154].

Polyrotaxane hydrogels for multimodal interaction. The mechanically interlocked polyrotaxane hydrogel pushes simultaneously on every performance axis that this review has traced (Figure 12E) [12]: the pulley effect of macrocycles sliding along polymer chains dissipates stress without accumulating damage, yielding skin-like softness (modulus ~8.5 kPa), ultrahigh stretchability (2450%), and cyclic stability over 10,000 loading cycles; the ionic network delivers 7.46 mS cm^−1^ conductivity; and as an epidermal electrode it acquires high-fidelity ECG and EMG signals at a signal-to-noise ratio above 42 dB even during motion, sustained over 24-hour sessions. A five-sensor finger array built on the platform demonstrates real-time gesture recognition with wireless robotic control—closing the loop from soft-matter sensing to remote actuation. The system epitomizes the review’s throughline: supramolecular architecture (Section 2), multimodal robustness (Section 4 and Section 5), and AI-mediated interaction (Section 6.2) integrated into one material platform.

The AI integration horizon. Across every application scenario surveyed above, the integration of hydrogel sensing with machine learning is no longer optional but structural. Deep-learning-assisted pipelines now translate raw hydrogel signals into sign-language sentences, cervical-spine posture assessments, Huntington’s disease screening indicators, and infant-movement analytics for intensive care [155,156,157,158,159,160]. The pattern is consistent: hydrogels supply compliant, multimodal, always-on signal acquisition; neural networks supply the pattern recognition that classical signal processing cannot deliver on nonstationary, crosstalk-laden soft-matter signals; and the combination unlocks application domains—clinical screening, gesture languages, prosthetic control—that neither technology reaches alone. The next frontier is on-device inference, in which lightweight models execute on the same flexible substrate as the sensor, eliminating the wireless bottleneck between sensing and interpretation and completing the sensor’s evolution from passive material to intelligent system.

## 7. Challenges and Perspectives

The preceding sections have traced hydrogel sensing from network chemistry to intelligent wearable systems, and the trajectory is unmistakably ascendant: working ranges that now exceed 2800%, thermopowers three orders of magnitude above electronic materials, healing efficiencies above 90% within minutes, gesture recognition above 92% accuracy, and operational windows spanning −80 to +80 °C. Yet each of these superlatives was demonstrated under controlled conditions on a laboratory timescale, and the gap between such demonstrations and certified, manufacturable, field-durable products remains the field’s central unsolved problem. This closing section examines the five grand challenges that define that gap—long-term stability, biological safety, scalable manufacturing, multimodal crosstalk, and energy autonomy—identifying for each the state of the art, the specific scientific obstacles, and the most promising research directions.

### 7.1. Long-Term Stability

The long-term stability challenges discussed here should be understood in the context of the material-design strategies reviewed in Section 2 and Section 5. Network architecture, solvent engineering, salt loading, deep eutectic solvents, and self-healing chemistry all determine how a hydrogel sensor responds to water loss, swelling, drying, freezing, chemical degradation, and conductivity drift. Eery property that makes hydrogels attractive—their water content, softness, and ionic conductivity—is a property that the environment actively degrades. Water evaporates under ambient conditions, freezing destroys network structure below 0 °C and even at constant temperature, hydrogels undergo slow aging: physical crosslinks coarsen, filler networks consolidate, and ionic distributions relax toward equilibrium, so that a sensor’s calibration drifts even when nothing appears to happen to it. The strategies reviewed in Section 5—binary solvents, salt loading, DES phases—extend survival from days to weeks and months, but each imposes costs: cosolvents plasticize and lose water slowly; salts deliquesce and drift dimensionally; DES phases weaken the hydrogen-bond networks that provide toughness [161]. The honest assessment is that no current formulation maintains full mechanical, electrical, and interfacial performance across a multi-month deployment in uncontrolled humidity, and the systems that come closest are composite formulations whose complexity raises the manufacturing challenges of Section 7.3. The most promising directions are self-adhesive encapsulation architectures that decouple the sensing surface from the bulk environment, atmospheric moisture-digesting zwitterionic skins that actively harvest ambient water to offset evaporation, and—most fundamentally—aging science: systematic, time-resolved characterization of what actually changes in service, which remains under-studied relative to the performance metrics of fresh samples. Until the field routinely reports 3–6 month continuous-wear data alongside day-one performance, long-term stability will remain the discipline’s first credibility gap. Under realistic operating conditions, hydrogel sensors face simultaneous challenges that are rarely captured by laboratory tests. Sweat introduces ions, changes pH, and can leach components from the gel. Friction against clothing and skin can abrade the surface and damage electrodes. Repeated bending, stretching, and compression impose mechanical fatigue. Temperature and humidity fluctuate during daily activities, and storage conditions may vary widely. A systematic evaluation of long-term stability requires quantitative reporting of several parameters. First, continuous-wear data over 3 to 6 months should be reported, including changes in resistance, capacitance, or thermovoltage over time. Second, accelerated aging tests should be described, including temperature cycling between minus 20 and plus 60 degrees Celsius, humidity cycling between 20 percent and 90 percent relative humidity, and repeated mechanical deformation. Third, calibration drift should be quantified as the percentage change in sensitivity or baseline signal per week or per month. Fourth, encapsulation and packaging strategies should be described in detail, including water vapor transmission rate, oxygen transmission rate, and adhesion to skin. Fifth, batch-to-batch variability in long-term stability should be reported for at least three independent fabrication batches. Without these quantitative data, it is impossible to compare the long-term stability of different hydrogel sensors or to predict their service lifetime in real wearable applications.

These stability challenges are directly linked to the material-design strategies discussed earlier. Covalent double networks provide mechanical integrity but may embrittle under repeated swelling and drying. Semi-interpenetrating networks decouple conductivity from stiffness but can suffer from long-term ion migration and humidity sensitivity. Organohydrogels and mixed-solvent gels suppress freezing and drying but require careful solvent selection to maintain conductivity and skin compatibility. Salt-loaded systems maintain ionic transport at low temperature but can deliquesce and drift dimensionally. Deep eutectic solvents and ionic liquids eliminate evaporative loss but may weaken hydrogen-bond networks and require additional dynamic crosslinking. Self-healing chemistries can restore electrical continuity after damage, but their long-term stability depends on bond exchange kinetics and environmental tolerance. Therefore, stability should not be treated as a separate problem; it is a direct consequence of material design choices, and future hydrogel sensors should be designed with stability as a first-class constraint from the outset.

### 7.2. Biocompatibility and Safety

The biological evaluation of hydrogel sensors lags their chemical sophistication. Individual components—PVA, alginate, gelatin, borate, LiCl—are each individually well characterized, but chronic skin contact introduces leachables whose joint toxicity is unexamined: unreacted acrylamide monomer (a documented neurotoxin), photoinitiator fragments, MXene oxidation products, ionic-liquid cations, and the degradation products of dynamic covalent bonds all migrate from the gel into the stratum corneum. For implantable applications, the stakes escalate: foreign-body response, fibrous encapsulation, and chronic inflammation have ended more hydrogel device programs than any mechanical failure [162]. The field’s evaluation practice is dominated by acute cytotoxicity assays on cell monocultures, which are necessary but profoundly insufficient: they say nothing about sensitization, irritation, genotoxicity, or the multi-year degradation profile that ISO 10993 certification demands for medical devices. Three directions are critical. First, standardized sub-chronic skin-contact studies under realistic wear protocols—sweat, friction, occlusion—conducted with the exact commercial formulations intended for deployment. Second, chemistry-by-design for safety: monomer-free photopolymerization, leaching-resistant ionic liquids, and MXene passivation layers that prevent oxidative release. Third, honest toxicological characterization of the novel components—2D materials, DES phases, liquid metals—whose biological profiles are still being established, so that the field does not repeat the pattern of building its platform on components whose safety profile emerges only after deployment at scale. A systematic evaluation of biocompatibility and degradation requires several specific data types. First, leachables should be identified and quantified. For each component, the migration rate into artificial sweat, phosphate-buffered saline, or cell culture medium should be measured using liquid chromatography mass spectrometry or inductively coupled plasma mass spectrometry. Second, chronic skin-contact studies should be conducted under realistic wear protocols, including sweating, friction, occlusion, and repeated bending. These studies should report skin irritation, sensitization, and barrier function over at least 4 weeks. Third, ISO 10993 tests should be performed according to the intended application. For skin-contact sensors, ISO 10993-1 [79], ISO 10993-5 [80], and ISO 10993-10 [81] are essential. For implantable sensors, ISO 10993-6 [83] and ISO 10993-11 [82] are also required. Fourth, degradation products should be characterized. The degradation rate, degradation products, and their toxicity should be reported for both in vitro and in vivo conditions. Fifth, the biological fate of novel components such as MXene, deep eutectic solvents, ionic liquids, and liquid metals should be studied, including biodistribution, accumulation, and clearance. Table 8 summarizes the biocompatibility and degradation concerns of representative components and recommends specific tests. Adopting these systematic evaluations will ensure that hydrogel sensors are safe for long-term wearable and implantable applications.

The biological safety of hydrogel sensors depends not only on the chemical identity of the components but also on contact duration and application scenario. In short-term skin-contact applications, the main concerns are irritation, sensitization, and acute cytotoxicity from residual monomers, organic solvents, crosslinkers, photoinitiator fragments, nanoparticles, MXene oxidation products, ionic-liquid cations, and other additives. Because exposure is intermittent and doses are generally low, these risks can often be mitigated by purification, monomer-free polymerization, low-toxicity solvents, and encapsulation. In long-term wearable applications, chronic exposure, sweat-induced leaching, friction, occlusion, and accumulation of degradation products become important. Sweat can extract unreacted species, change pH, and accelerate hydrolysis of dynamic covalent bonds. The resulting leachates may cause contact dermatitis or systemic exposure. For implantable applications, the risks escalate to foreign-body response, fibrous encapsulation, chronic inflammation, thrombosis, and systemic toxicity. Long-term degradation products must be characterized, and their biodistribution, accumulation, and clearance must be evaluated. Leachables and degradation products should be characterized using liquid chromatography mass spectrometry and inductively coupled plasma mass spectrometry, and testing should be performed on the final commercial formulation rather than on individual components alone.

**Table 8:** Biocompatibility and degradation concerns of representative components in hydrogel sensors.

Component	Leachables or Degradation Products	Concerns and Mitigation	Recommended Tests	References
PAM and PAA matrix	Residual acrylamide monomer	Neurotoxicity, skin sensitization, and carcinogenicity; mitigated by monomer-free polymerization, extensive purification, and radical scavengers	ISO 10993-5 [80]; ISO 10993-10 [81]; LC-MS leachate analysis	[161]
Photoinitiators	Radical fragments and benzaldehyde derivatives	Cytotoxicity, irritation, and genotoxicity; mitigated by using LAP instead of Irgacure 2959, low-toxicity initiators, and inert blanketing	ISO 10993-5 [80]; ISO 10993-3 [163]; extraction tests	[57,58,59]
MXene	Titanium oxide particles and fluoride residues	Oxidative degradation, dose-dependent cytotoxicity, and pulmonary inflammation; mitigated by ascorbate scavengers, PVA passivation, and lyophilization for storage	ICP-MS; cell viability; chronic exposure; ISO 10993-5 [80]	[41,42,43]
Ionic liquids	Imidazolium cations and halide anions	Ecotoxicity, skin permeation, and mitochondrial toxicity; mitigated by biocompatible cations, polymer-supported ionic liquids, and immobilization	OECD 439 [164]; ISO 10993-10 [81]; cytotoxicity; sensitization	[115,116,117,118]
Deep eutectic solvents	Choline chloride, urea, and imidazole	Long-term skin contact unclear and potential irritation; mitigated by encapsulation, screening of hydrogen-bond donors, and dilution	ISO 10993-10 [81]; leachate analysis; patch tests	[114]
Borate esters	Borate ions and diol exchange products	High-dose reproductive toxicity and irritation; mitigated by covalent anchoring, low leaching, and alternative dynamic bonds	Extraction; ICP-OES; ISO 10993-10 [81]	[119,120,121]
Liquid metals	Gallium ions, indium ions, and nanoparticles	Long-term biodistribution unclear and oxidative stress; mitigated by encapsulation, surface oxide control, and alloy optimization	ICP-MS; chronic implantation; ISO 10993-6 [83]	[132]
Dynamic covalent bonds	Schiff base hydrolysis products and acylhydrazone fragments	Unknown chronic toxicity and pH-dependent release; mitigated by stable bond selection, pH buffering, and encapsulation	LC-MS; ISO 10993-5 [80]; degradation profiling	[125,126,127]
Carbon nanotubes	Residual metal catalysts and carbon nanoparticles	Pulmonary inflammation and oxidative stress; mitigated by purification, surface functionalization, and encapsulation	ISO 10993-5 [80]; ISO 10993-11 [82]; ICP-MS	[128,129,130]
Silver nanowires	Silver ions and nanoparticles	Argyria, cytotoxicity, and environmental persistence; mitigated by encapsulation, low-loading, and alternative conductors	ICP-MS; ISO 10993-5 [80]; ISO 10993-10 [81]	[101]

### 7.3. Scalable Synthesis and Manufacturing

The laboratory synthesis of high-performance hydrogel sensors is artisanal: multi-step protocols with manual timing, batch-to-batch variability that academic papers routinely report as a few percent but industrial quality control would measure differently, and form factors that assume a skilled operator rather than a production line. 3D printing—one of the most widely explored fabrication advances—transfers awkwardly to scale: printer throughput is measured in centimeters per hour, photocurable inks sacrifice the toughness of thermodynamically equilibrated networks, and the print-defect statistics of Section 2.3 (layer anisotropy, nozzle clogging, residence-time windows) compound with material variability [165]. Roll-to-roll processing, screen printing, and injection molding offer the throughput that printing cannot, but they demand rheological formulation windows that the field’s most advanced chemistries—polyrotaxane pulley networks, dynamic covalent triple systems—do not yet occupy. The manufacturing problem is therefore not a single missing technology but a translation gap: laboratory formulations are optimized for performance metrics measured on day one, not for the process windows, inline metrology, and statistical process control that manufacturing imposes. The most promising developments are closed-loop printing systems with inline electrical and rheological inspection, digitally formulated ink libraries whose properties are certified before printing, and design-for-manufacture studies that explicitly trade peak performance for reproducibility. Until such approaches mature, the field should expect the familiar pattern: extraordinary laboratory demonstrations, thin industrial follow-through, and the persistent question of why the best published sensors cannot be purchased.

### 7.4. Multimodal Crosstalk

Section 3.6 identified crosstalk as the physics-level obstacle to interpretation, and the systems-level consequences deserve equal weight. A deployed multimodal sensor produces streams whose values are entangled: strain readings contaminated by temperature drift, humidity signals confounded by mechanical artifacts, electrophysiology corrupted by motion. The three decoupling layers reviewed there—materials, device stacks, and algorithms—each advance, but none yet solves the problem at deployment timescales, where the crosstalk manifold itself drifts as the sensor ages [166]. The unresolved questions are quantitative: What crosstalk floor is achievable in principle? How much of the entanglement is invertible by any algorithm, and how much is information destroyed at acquisition? And how do learned decoupling models transfer across users, wear sites, and weeks of aging, when the training data that fit them no longer describes the device? The research directions follow: information-theoretic analysis of soft-matter sensing that establishes the decoupling limits; longitudinal datasets—publicly archived, spanning months of continuous wear—that allow the community to study drift itself; and self-supervised adaptation schemes in which models recalibrate from unlabeled in-service signals. Crosstalk is the least glamorous of the five challenges, but it may be the most consequential: every application domain of Section 6 is ultimately gated by the reliability of signal interpretation, and interpretation is precisely where soft matter underperforms rigid electronics. Standardized benchmarking protocols, as proposed in Section 3.8 and summarized in Table 3, are therefore essential for comparing decoupling performance across studies. Signal processing and readout electronics, as discussed in Section 3.9, are equally essential for reliable decoupling and interpretation.

### 7.5. Energy Autonomy

Self-powered sensing was presented in Section 3 as a solved mechanism problem—TENGs and iTE devices demonstrably harvest ambient energy—and as a system problem it is far from solved. Harvest budgets are microwatts; wireless transmission consumes milliwatts; the six-order-of-magnitude mismatch confines self-powered systems to duty-cycled operation at very low transmission rates, or to wired readout, which contradicts the premise of distributed wearable sensing [167]. Energy storage closes part of the gap—flexible supercapacitors and thin-film batteries buffer harvested energy for burst transmission—but flexible storage introduces its own calendar-life and cycle-life limitations that compound, rather than resolve, the system’s aging problem. Near-field communication sidesteps transmission power by harvesting the reader’s field, and the battery-free wound sensor of Section 6.1 demonstrates the approach’s elegance, but NFC range is centimeters, which re-tethers the user to a reader device. The realistic near-term architecture is hybrid: energy harvesting extends battery life from days to months, rather than eliminating batteries outright. The medium-term hope lies in ultralow-power inference (Section 6.3) that reduces what must be transmitted from raw signals to decisions—kilobits instead of megabits—collapsing the energy budget at the communication layer. The long-term question is whether the energy density of ambient mechanical and thermal sources near the human body can ever sustain continuous sensing-communication loops, or whether hydrogel wearables will remain, by thermodynamic necessity, event-driven devices.

### 7.6. Future Perspectives

The five challenges share a common structure: each marks a frontier where the soft matter that makes hydrogel sensors valuable—compliant, hydrated, ionically conducting, chemically dynamic—is the same soft matter that undermines stability, safety, manufacture, interpretation, and power. The resolution will not come from abandoning softness but from engineering it deliberately: chemical economies in which every component serves multiple defensive functions; safety co-designed into monomer selection rather than audited afterward; manufacturing windows respected at formulation time; crosstalk treated as a first-class design constraint; and energy budgets engineered at the system level from the outset. The convergence already visible in the literature—polyrotaxane gels that integrate mechanics, electrophysiology, and robotic control; all-soft ECL systems that cost less than the bandage that holds them; machine-learning pipelines that turn nonstationary soft-matter noise into clinical signal—suggests the trajectory. The next decade will determine whether hydrogel sensors complete the transition from remarkable materials to reliable infrastructure, and the evidence assembled in this review suggests that the materials science is ready; what remains is the systems engineering, the safety science, and the longitudinal evidence that certification and clinical adoption demand. Future progress also requires community-wide adoption of standardized reporting and benchmarking protocols, such as the checklist proposed in Section 3.8 and Table 3, without which cross-study comparison will remain unreliable. Signal processing and readout electronics, as discussed in Section 3.9, are also critical for translating soft-matter signals into reliable wearable information. The following section discusses the environmental impact and sustainability of hydrogel sensors. The final section then summarizes the main conclusions of this review and outlines the most important directions for future research. Systematic evaluation of biocompatibility, toxicity, degradation data, and long-term stability, as discussed in Section 7.2 and summarized in Table 8, is therefore essential for translating hydrogel sensors from laboratory demonstrations to certified wearable and implantable products.

### 7.7. Environmental Impact and Sustainability

The environmental impact and sustainability of hydrogel sensors have received far less attention than their sensing performance, even though many hydrogel components are described as green or sustainable. A complete sustainability assessment must consider the entire life cycle, including raw material extraction, synthesis, fabrication, deployment, and end-of-life disposal.

Bio-based polymers such as cellulose, alginate, gelatin, and hemicellulose offer renewable feedstocks and can reduce dependence on petroleum-derived monomers. However, renewability alone does not guarantee low environmental impact. The cultivation, extraction, purification, and transportation of bio-based feedstocks consume energy and water and can generate agricultural or processing waste. Life-cycle assessment is therefore necessary to compare bio-based and synthetic hydrogels on a consistent functional basis.

Synthetic hydrogels and sustainability. Synthetic hydrogels such as polyacrylamide, polyacrylic acid, and polyvinyl alcohol dominate the literature because their chemistry is tunable and their performance is reproducible. However, their sustainability profile is complex. Their monomers are often petroleum-derived, and residual monomers such as acrylamide are toxic. Their synthesis can require organic solvents, high energy input, and multi-step purification. Their disposal is also problematic: crosslinked networks are difficult to degrade, and they can persist in the environment for years. Several strategies can reduce the environmental impact of synthetic hydrogels. First, monomer selection can be shifted toward bio-based or less toxic alternatives. Second, polymerization can be performed in water or green solvents, and photoinitiators can be selected for low toxicity. Third, crosslinking can be designed to be reversible or cleavable, enabling controlled degradation after service life. Fourth, recycling can be improved through design for disassembly and reduction in material diversity. Fifth, life-cycle assessment should be used to compare synthetic and bio-based hydrogels on a consistent functional basis. Finally, reporting sustainability data alongside sensing performance would make trade-offs transparent and support informed material selection.

Biodegradability must be balanced against durability. Sensors designed to degrade rapidly may lose mechanical and electrical performance during use, especially in humid or biologically active environments. Conversely, sensors designed for long-term stability may persist in the environment for years after disposal. Programmable or controlled degradation offers a potential solution, in which the sensor remains stable during its intended service life and then degrades under a specific trigger such as pH, temperature, enzymes, or light.

Green solvents and deep eutectic solvents are often presented as environmentally friendly alternatives to conventional organic solvents. However, their environmental profile depends on the specific hydrogen-bond donor and acceptor, the synthesis route, the energy required for preparation, and their ecotoxicity and persistence after release. Choline-based deep eutectic solvents are generally considered less toxic than imidazolium-based ionic liquids, but systematic ecotoxicological data remain limited. Ionic liquids can be persistent and mobile in water, and some imidazolium cations exhibit aquatic toxicity. Therefore, solvent selection should be based on measured environmental data rather than on the label of green solvent.

Nanomaterials used in hydrogel sensors also raise environmental concerns. MXene can oxidize and release titanium oxide particles or fluoride residues. Liquid metals can release gallium or indium ions. Carbon nanotubes, carbon nanoparticles, and silver nanowires can persist in the environment and may cause ecotoxicity. These materials must be evaluated not only for human biocompatibility but also for environmental fate, bioavailability, and long-term ecosystem impact.

Electronic waste is another critical issue. Wearable sensors often contain electrodes, interconnects, encapsulation layers, batteries, and wireless communication components. Even a hydrogel-based sensor is rarely composed of a single material. Multi-material design complicates recycling and increases end-of-life burden. Design for disassembly, reduction in material diversity, and replacement of non-recyclable components with biodegradable or recyclable alternatives can substantially reduce environmental impact.

Recycling strategies for hydrogel sensors include mechanical recycling, chemical recycling, and biological recycling. Mechanical recycling can recover polymers and fillers, but contamination and crosslinking can degrade material quality. Chemical recycling can break down networks into monomers or oligomers, but it requires energy and solvents. Biological recycling can degrade bio-based hydrogels under controlled conditions, but it must be adapted to the specific polymer and application. A circular design approach should consider recyclability at the material selection stage rather than after the device is fabricated.

Life-cycle assessment provides a quantitative framework for evaluating environmental impact. Standard methods such as ISO 14040 [168] and ISO 14044 [169] define principles, framework, requirements, and guidelines for life-cycle assessment. A complete assessment should define the functional unit, system boundary, inventory data, impact categories, and interpretation method. For hydrogel sensors, relevant impact categories include global warming potential, water consumption, energy demand, ecotoxicity, and resource depletion. Without such assessments, claims of sustainability remain qualitative and difficult to compare.

Regulations also shape sustainable design. RoHS restricts hazardous substances in electrical and electronic equipment. REACH regulates chemicals in the European Union. WEEE addresses waste electrical and electronic equipment. These regulations do not yet specifically target hydrogel sensors, but they establish principles that future wearable sensors should follow. Proactive compliance can reduce regulatory risk and improve market acceptance.

Sustainable design principles for hydrogel sensors can be summarized as follows. First, reduce material diversity and avoid unnecessary components. Second, prioritize bio-based, biodegradable, and low-toxicity materials where they do not compromise performance. Third, design for disassembly and recycling. Fourth, minimize energy and water consumption during fabrication. Fifth, extend service life through encapsulation, self-healing, and stable chemistry. Sixth, quantify environmental impact through life-cycle assessment. Seventh, report sustainability data alongside sensing performance so that trade-offs are transparent.

In summary, environmental impact and sustainability should be treated as first-class design constraints for hydrogel sensors rather than as post hoc considerations. The field must move from claiming sustainable materials to demonstrating sustainable systems, including measured degradation, recycling, and life-cycle data. The final section summarizes the main conclusions of this review and outlines the most important directions for future research.

### 7.8. Barriers to Commercialization

Despite rapid progress in laboratory demonstrations, hydrogel sensors have not yet achieved widespread commercial translation. Several barriers are responsible.

First, long-term stability remains insufficient. Hydrogels are vulnerable to dehydration, freezing, swelling, and gradual aging, and no current formulation maintains full mechanical, electrical, and interfacial performance across a multi-month deployment in uncontrolled humidity. Encapsulation and packaging strategies can extend lifetime, but they add cost and complexity and may compromise skin conformity.

Second, biocompatibility and safety data are incomplete. Chronic skin-contact studies, leachables analysis evaluation are often performed on individual components rather than on final commercial formulations. Residual monomers, photoinitiator fragments, MXene oxidation products, ionic-liquid cations, and degradation products of dynamic covalent bonds can migrate into the skin, and their joint toxicity is rarely examined. For implantable applications, foreign-body response, fibrous encapsulation, and chronic inflammation remain major risks.

Third, manufacturing reproducibility is limited. Laboratory synthesis is artisanal, with manual timing and batch-to-batch variability that industrial quality control would not accept. Rheological properties, electrical conductivity, and mechanical performance can vary significantly between batches, and standardized ink characterization protocols are still lacking.

Fourth, scalable fabrication methods are still immature. 3D printing offers customization but low throughput, while roll-to-roll processing, screen printing, and injection molding require rheological formulation windows that many advanced hydrogel chemistries do not yet occupy. Print defects such as layer anisotropy, nozzle clogging, and residence-time windows compound with material variability.

Fifth, calibration and signal interpretation remain difficult. Hydrogel sensors suffer from crosstalk, drift, and nonstationarity, and multimodal decoupling algorithms are not yet robust across users, wear sites, and aging. Without reliable calibration, clinical and consumer applications cannot trust the measured values.

Sixth, energy autonomy is unresolved. Harvest budgets are microwatts, wireless transmission consumes milliwatts, and flexible energy storage introduces additional calendar-life and cycle-life limitations. Self-powered systems therefore remain confined to duty-cycled operation at very low transmission rates, or to wired readout.

Seventh, standardization is lacking. Without common definitions for sensitivity, response time, hysteresis, self-healing efficiency, biocompatibility, and long-term stability, cross-study comparison and regulatory approval remain difficult. Benchmarking protocols are only beginning to emerge.

Eighth, cost and user acceptance are uncertain. High-performance hydrogels often require expensive fillers, complex synthesis, or specialized encapsulation, and users may resist devices that require frequent recalibration or maintenance. Adhesion, comfort, and breathability also affect long-term wearability.

Overcoming these barriers will require coordinated progress in materials chemistry, device engineering, manufacturing science, regulatory science, and human factors research. The most promising near-term strategy is to prioritize applications where hydrogel sensors offer unique advantages, such as skin-conformal biosensing, wide-temperature strain sensing, and self-powered tactile interfaces, while continuing to improve stability, safety, and manufacturability.

## 8. Conclusions and Outlook

This review has traced hydrogel and organogel sensors from network synthesis to wearable deployment. The field has achieved remarkable progress, including working ranges exceeding 2800 percent, ionic thermopowers above 20 millivolts per kelvin, self-healing efficiencies above 90 percent within minutes, gesture recognition accuracy above 92 percent, and operational windows from minus 80 to plus 80 degrees Celsius. However, the gap between laboratory demonstrations and certified, manufacturable, field-durable products remains the central challenge.

Six sensing mechanisms have been compared in this review, namely piezoresistive, capacitive, triboelectric, ionic thermoelectric, electrochemical, and multimodal decoupling. Their performance characteristics are summarized in Table 1. No single mechanism dominates. Piezoresistive sensing maximizes sensitivity per unit complexity, capacitive sensing offers stability and low power, triboelectric and ionic thermoelectric modalities enable self-powered operation, electrochemical sensing reaches molecular analytes, and multimodal decoupling provides the design discipline needed for realistic deployment. The most capable systems combine two or more mechanisms in integrated stacks.

Mechanical robustness and sensitivity enhancement have advanced through double-network energy dissipation, physical crosslinking, nanofiller reinforcement, Hofmeister-effect engineering, material-level sensitivity strategies, and structural engineering. However, these two axes remain coupled in practice, because the microstructural features that amplify signals are frequently the same features that concentrate stress. Future designs should be judged by the Pareto frontier they trace across mechanics, sensitivity, stability, and lifetime.

Self-healing chemistry and environmental adaptation are essential for long-term operational stability. Hydrogen-bond and borate ester synergism, dynamic metal-ligand coordination, multiple dynamic covalent bonds, and nanocomposite-assisted healing each offer distinct trade-offs between healing speed, recovered strength, and environmental tolerance. Anti-freezing strategies based on solvent replacement, salt incorporation, deep eutectic solvents, and ionic liquids extend operation from subzero temperatures to elevated temperatures, but each strategy imposes costs in terms of conductivity, mechanical properties, or biocompatibility.

A systematic evaluation of long-term stability and biocompatibility is urgently needed. As discussed in Section 7.1 and Section 7.2, future studies should report 3- to 6-month continuous-wear data, accelerated aging tests, temperature and humidity cycling, calibration drift, leachables identification, chronic skin-contact studies, degradation product characterization, and the bi ological fate of novel components. Table 8 summarizes the biocompatibility and degradation concerns of representative components and recommends specific tests. Without these data, cross-study comparison and regulatory approval will remain difficult.

Standardized reporting and benchmarking protocols are also essential. As proposed in Section 3.8 and summarized in Table 3, future studies should report gauge factor definition and strain or pressure range, linearity and coefficient of determination, response and recovery time definitions, hysteresis, cyclic stability, temperature and humidity conditions, self-healing mechanical and electrical efficiency, adhesion strength, biocompatibility evaluation, long-term stability, multimodal crosstalk, an statistical reporting. Adopting this minimum reporting checklist would greatly improve reproducibility and enable meaningful comparison across hydrogel sensor platforms.

Application frontiers continue to expand. Hydrogel and organogel sensors are being deployed in health monitoring biosensing, electronic skin, human–machine interaction, AI-assisted gait analysis, exoskeleton-assisted rehabilitation, underwater operation, osmotic sweat harvesting, and polyrotaxane-based multimodal interaction. Across all these scenarios, the integration of hydrogel sensing with machine learning is becoming structural rather than optional. Deep-learning pipelines translate raw hydrogel signals into gesture recognition, sign-language sentences, gait diagnoses, and rehabilitation assessment. The next frontier is on-device inference, in which lightweight models execute on the same flexible substrate as the sensor, eliminating the wireless bottleneck between sensing and interpretation.

Several grand challenges remain. First, long-term stability requires systematic reporting of continuous-wear data, accelerated aging, and calibration drift. Second, biocompatibility and safety demand leachables identification, chronic skin-contact studies, and degradation product characterization. Third, scalable manufacturing must address process windows, inline metrology, and batch-to-batch reproducibility. Fourth, signal processing and readout electronics, as discussed in Section 3.9, are essential for wearable deployment because they determine power consumption, form factor, noise, and the reliability of multimodal decoupling. Fifth, multimodal crosstalk requires materials-level, device-level, and algorithm-level decoupling strategies with quantified residual error. Sixth, energy autonomy remains limited by the microwatt-scale harvest budget and milliwatt-scale transmission demand, motivating hybrid harvesting and ultralow-power inference. Seventh, environmental sustainability, as discussed in Section 7.7, calls for bio-based polymers, biodegradable encapsulation, recycling strategies, and life-cycle assessment.

The next decade will determine whether hydrogel sensors complete the transition from remarkable materials to reliable infrastructure. The materials science is ready. What remains is the systems engineering, the safety science, the standardization effort, and the longitudinal evidence that certification and clinical adoption demand.

## Figures and Tables

**Table 5:** Comparison of mechanical and electrical healing performance of representative self-healing hydrogels.

Healing Chemistry	Healing Time and Conditions	Mechanical and Electrical Healing Efficiency	Number of Healing Cycles and Recovery	References
Hydrogen-bond and borate ester synergism	Seconds to minutes at room temperature; contact required	Mechanical healing efficiency moderate; electrical healing efficiency high	Several cycles; mechanical recovery moderate; electrical recovery rapid	[120,121,122]
Dynamic metal-ligand coordination	Minutes to hours at room temperature; metal ions required	Mechanical healing efficiency high; electrical healing efficiency moderate to high	Multiple cycles; mechanical recovery high; electrical recovery moderate	[123,124,125]
Multiple dynamic covalent bonds	Minutes; pH or thermal trigger required	Mechanical healing efficiency high; electrical healing efficiency moderate to high	Multiple cycles; mechanical recovery high; electrical recovery moderate	[126,127,128]
Nanocomposite-assisted healing	Minutes; contact required; filler network reconnection	Mechanical healing efficiency moderate; electrical healing efficiency high	Several cycles; mechanical recovery moderate; electrical recovery rapid	[129,130,131]

## Data Availability

No new data were created or analyzed in this study.
